# Acoustic markers of emotional expression in professional lyrical singing

**DOI:** 10.3389/fpsyg.2026.1880734

**Published:** 2026-07-24

**Authors:** Carlos Hugo C. Del Valle

**Affiliations:** Universidad de Salamanca, Salamanca, Spain

**Keywords:** ecological validity, emotional induction, performance science, professional sopranos, singing voice, source-filter theory, 4E/5E cognition

## Abstract

**Introduction:**

This study examines the extent to which induced emotions (joy versus anger) modulate the acoustic architecture of professional lyrical sopranos. Fundamentally aligned with the 4E/5E paradigm of cognitive science, this research aims to analyze the extent to which induced affect reconfigures the vocal system during performance. Grounded in Non-Linear Source-Filter Theory, the study evaluates the functional interaction between glottal source dynamics (*F*_0_, SPL, and vibrato) and vocal filter strategies (formants *F*_1_ to *F*_5_).

**Methods:**

Three professional Category 2.1 soprano singers underwent an emotional induction protocol using validated film fragments to establish conditions of affective congruence and incongruence while performing selected musical excerpts. Standardized multi-parametric quantitative acoustic tracking was conducted to isolate individual harmonic profiles and verify upper formant configurations.

**Results:**

The effective induction of joy fosters resonance optimisation through efficient “*R*_1_:*f*_0_ tuning” and a marked expansion of the vowel space (*F*_1_ - *F*_2_ gap). In Singer 1, joy facilitated a precise alignment of *F*_3_ and *F*_5_ during the central vibrato cycle, clustering the Singer’s Formant to enhance spectral brightness and “squillo”. Conversely, induced anger prioritises physiological constriction of the vocal tract and increased glottal adduction—characterised by pronounced elevations in SPL and heightened subglottic pressure. In Singers 2 and 3, although the distances between upper formants remained broad, they maintained vocal projection through an intensified Singer’s Formant Cluster (SFC) achieved via more efficient excitation of higher resonances.

**Conclusion:**

These findings suggest that authentic emotional states physically and involuntarily optimise the singer’s acoustic output, whereas conflicting affect degrades fine motor technical stability. The results substantiate that classical performance is an enactive process of embodiment, offering key implications for voice pedagogy through explicit parameter modulation training and real-time acoustic feedback systems.

## Introduction

The capacity of music to express emotions is well-documented in the literature (Juslin and Västfjäll, 2008; Scherer and Coutinho, 2013). Singing, in particular, constitutes a primary modality of musical expression. Much like speech, singing allows for the communication of complex emotions through acoustic cues (Eerola and Vuoskoski, 2013; Escoffier et al., 2013; Juslin and Laukka, 2003; Scherer et al., 2015). While emotional transmission in both domains relies on similar acoustic parameters, recent studies suggest that the perceived intensity differs substantially between them (Livingstone et al., 2013).

In the training of a singer, the rigorous focus on technical proficiency often marginalizes emotional pedagogy, frequently failing to achieve the desired interpretive depth (Lehmann et al., 2007). Accordingly, vocalists must understand how emotional expression modulates the voice to achieve communicative efficacy without compromising acoustic sonority. Another important aspect is the authenticity of vocal expression, which remains a subject of intense debate regarding the acoustic and perceptual distinctions between genuine and simulated affect (Russell et al., 2003; Scherer, 2003; Vogt and André, 2005). Authors like Juslin (2013) commented that one can both perceive and express emotion without actually having an emotion. Other authors (Gabrielsson, 2001; Hodges, 2010) have proposed a distinction between perceived expressions of emotion and felt expressions of emotion, the difference being that perceived emotions can be recognized from music without necessarily feeling them, while felt emotions are actual emotions being induced in the listener by the music.

Emotional expression in the singing voice is operationalized through measurable acoustic parameters characterized via spectral analysis. Singers convey affect through subtle modulations of fundamental frequency (F_0_), temporal structure, intensity, and timbre (Juslin and Laukka, 2003, 2004; Sundberg et al., 2013). Consequently, stabilizing baseline variables such as F_0_ and tempo facilitates the systematic analysis of vowel-specific variations during emotional transmission (Coutinho et al., 2014), as performers utilize minor fundamental frequency (F_0_) fluctuations to reinforce their interpretive intent.

Considerable research has investigated the acoustic parameters underlying emotional expression in singing (Bachorowski and Owren, 2003; Eyben et al., 2015; Guzman et al., 2013; Scherer et al., 2017). Numerous investigations (Kamiloğlu et al., 2020; Scherer et al., 2015; Scherer and Sundberg, 2017) have delineated emotion-specific acoustic profiles by contrasting expressive singing against neutral vocal baselines. Sundberg et al. (1995) utilized a comparative methodology, contrasting simulated concert interpretations with emotionally neutral renderings of opera and Lieder excerpts. Expert perceptual analysis confirmed that high-arousal emotional portrayals (e.g., joy, anger) were acoustically distinguished by significantly higher Sound Pressure Levels (SPL), accelerated tempi, and increased dynamic variability relative to low-arousal expressions (e.g., sadness, tenderness). Beyond descriptive research, Hakanpää (2022) demonstrated that targeted training in parameter modulation significantly enhances emotional expressivity. Post-intervention results confirmed that regulating diverse acoustic parameters facilitates a more systematic and precise communication of affective intent during performance.

To provide an organised exposition of the markers associated with emotional expression, this study is grounded in the “Non-Linear Source-Filter Theory (Titze, 2008), which conceptualises sung voice production as a functional interaction between two primary physical components. Firstly, the glottal source provides the acoustic “raw material” through vocal fold vibration, determining pitch and intensity via parameters such as Fundamental Frequency (*F*_0_), the Harmonic series, Vibrato, and SPL. Secondly, the vocal tract filter acts as a resonator that selectively amplifies or attenuates these harmonics, a process primarily measured through Formants (*F*_1_–*F*_4_). This interaction constitutes the physiological foundation for emotional transmission, as professional singing optimises the source-filter relationship through specific strategies to convey affect—most notably “*R*_1_:*f*_0_ tuning” to maximise resonance and the Singer’s Formant Cluster (SFC) to ensure vocal projection and emotional “ring”—meaning that while the source dictates the signal’s energy, the filter transforms it to provide the specific timbral and emotional identity required by the induction. Importantly, this interaction between the vocal source harmonics and the tract formants is crucial not only for timbral definition, but also plays a pivotal role in central auditory integration and neural processing (Saus et al., 2025).

In relation to the first physical component, the glottal source acts as the primary sound generator. Within this domain, the *F*_0_ is the lowest frequency generated by the vocal folds and corresponds to the base tone of the voice (Pulkki and Karjalainen, 2015). In the singing voice, while *F*_0_ defines the musical pitch, its variability is what truly encodes emotional information (Owren and Bachorowski, 2007; Scherer et al., 2017). In this regard, a substantial body of research (Juslin and Laukka, 2003; Laukka et al., 2005; Paquette and Peretz, 2014; Scherer, 2003) has highlighted *F*_0_––related acoustic parameters as robust markers of emotional arousal and physiological activation, particularly for high-energy affects such as joy and anger. Specifically, Juslin and Laukka (2003) established that a low *F*_0_ is consistently associated with sadness, while Murray and Arnott (1993) demonstrated that high-arousal emotions typically elevate *F*_0_, whereas lower values characterise sadness and tenderness. Furthermore, elevated mean *F*_0_, a greater range, and rapid frequency fluctuations are indicative of high physiological arousal. Conversely, Scherer (1986) noted that sadness is characterised by reduced *F*_0_ variability, resulting in a perceptually monotonous quality. Quantifying the *F*_0_ from a singing voice is always an approximation, as the signal rarely remains stable, even on a single pitch. As noted, the *F*_0_ establishes the base tone, while harmonics and formants provide spectral richness; thus, the interaction between these components is what renders the singing voice uniquely expressive.

Harmonics are a variable of interest because they are responsible for the richness and tonal complexity of the voice (Sundberg, 2001a, 2001b). A single voiced sound is a complex periodic wave consisting of a *F*_0_ and a series of additional frequencies called harmonics, which are always integer multiples of the *F*_0_. These are generated by the quasi-periodic pulsating glottal airflow, which is modulated by the rapid opening and closing of the vocal folds. While their initial energy is determined by this glottal source flow, their relative amplitude in the radiated sound is shaped by the resonances of the vocal tract. The specific distribution of energy across this harmonic series is what defines the unique timbre and identity of each voice.

Vibrato is a fundamental expressive resource, volitionally modulated by performers to convey specific artistic and emotional intent. Acoustically, it is defined as the periodic and systematic oscillation of F_0_ and/or amplitude (Liu et al., 2022). Holmes (2013) identifies three core parameters: vibrato rate (frequency in Hz), vibrato extent (frequency modulation in cents/semitones), and regularity (the stability of modulations). While standard rates typically range between 4–7 Hz with an extent of 0.1–0.5 semitones, these values shift according to emotional arousal. High-arousal states, such as joy and anger, are characterised by accelerated rates (>7 Hz), whereas low-arousal emotions, including sadness and tenderness, are linked to slower rates (<5 Hz) or even the absence of vibrato. Vibrato extent and stability further signal emotional intensity and balance; specifically, a wide extent indicates dramatic intensity, while restricted vibrato conveys serenity (Holmes, 2013). Numerous investigations (Dromey et al., 2015; Jansens et al., 1997; Konishi et al., 2002; Langeheinecke et al., 1999; Sundberg et al., 1995) have identified emotion-specific variations. Specifically, joy is associated with a wide and stable vibrato, whereas anger is distinguished by an accelerated vibrato rate, and elevated F_0_ driven by increased laryngeal hyperfunction, alongside reduced stability (Jansens et al., 1997; Konishi et al., 2002). Heightened vibrato irregularity, characterised by abrupt cycle-to-cycle variations, conveys a strained quality or perceived aggressiveness (Liu et al., 2022). Finally, Holmes (2013) demonstrated that increased emotional intensity induces greater rate inconsistency, aligning with research linking heightened affective states to a trembling vocal quality.

Finally, Sound Pressure Level (SPL) —the acoustic correlate of loudness— is one of the most salient parameters in the encoding and decoding of affect in the singing voice. Hakanpää et al. (2019) demonstrated that SPL and the spectral distribution of energy constitute the two most characteristic indicators of emotional voice quality. High-arousal emotions, such as joy and anger, are consistently characterised by significantly higher SPL than low-arousal states, such as sadness or tenderness (Hakanpää et al., 2021; Livingstone et al., 2014; Scherer et al., 2017; Sundberg, 2021). Notably, as many acoustic descriptors are intrinsically tied to SPL, isolating emotion-specific modulations from intensity-dependent changes remains methodologically complex. For instance, Hakanpää et al. (2021) reported that *F*_0_ significantly impacts overall SPL, with intensity typically increasing in tandem with frequency.

Regarding the second physical component, the vocal tract filter acts as a primary resonator where resonance frequencies constitute another critical variable. These represent the natural frequencies of the vocal tract which, when excited by a sound source, produce formants; these are defined as spectral peaks that represent the concentration of acoustic energy at specific frequencies (Story, 2016; Titze, 2015). Technically, formants result from resonances of the vocal tract acting as bandpass filters that selectively reinforce certain harmonics produced by the vocal folds. In singing, the resonance frequencies can be modulated by physiological adjustments such as tongue position, jaw height, lip aperture, and velum configuration. These vocal tract modifications shift the resonance frequencies, thereby allowing certain harmonics to be amplified when they coincide with those resonances. The harmonics, generated at the glottal source, interact with the vocal tract resonances; the closer a harmonic is to a resonance frequency, the more it is acoustically reinforced, appearing as a formant in the radiated spectrum. Consequently, a primary objective for singers is to synchronise resonance frequencies with specific harmonics through precise articulatory modifications (Sundberg, 2021).

In vocal acoustics, five primary formant frequencies are typically analysed to define timbre, which arises from the filtering of the glottal source by vocal tract resonances. The F_0_ generally ranging between 260 and 1,000 Hz, represents the rate of vocal fold vibration and is distinct from these resonances. Timbre and vowel identification depends primarily on the first two formants (F_1_ and F_2_): F_1_ is modulated by jaw opening and tongue height (250–1,400 Hz), while F_2_ reflects the anteroposterior tongue position (800–2,500 Hz). Goudbeek et al. (2009) investigated how emotional dimensions —valence, arousal, and power— reconfigure these articulatory parameters across various vowels. Their findings revealed that high-arousal emotions, such as joy and anger, significantly increase *F*_1_ values due to greater mouth opening, while positive emotions like joy are associated with higher *F*_2_ values through fronted tongue articulation. Furthermore, high vocal control was shown to expand the vowel space, enhancing the spectral differentiation between *F*_1_ and *F*_2_.

Two distinct resonant strategies facilitate acoustic projection and clarity in the lyrical voice by optimising the interaction between the glottal source and the vocal tract filter. The first, “*R*_1_:*f*_0_ tuning” (or resonance tuning), involves the systematic alignment of the first vocal tract resonance (R_1_) with the F_0_ or its lower harmonics to maximise vocal power and efficiency (Benade, 1990; Garnier et al., 2010). This phenomenon is crucial for acoustic reinforcement, particularly in notes where the F_0_ (or its harmonics, such as 2*F_0_ o 3*F_0_) approaches the frequency of R_1_. The second mechanism, the SFC, entails the convergence of upper formants (*F*_3_–*F*_5_) within the 2.5–4 kHz region (Sundberg, 1974, 2001a, 2001b). Physiologically mediated by laryngeal lowering and pharyngeal expansion, the SFC generates a prominent spectral peak that enhances perceived brightness and enables the voice to project over dense orchestral accompaniment (Granqvist et al., 2003; Titze, 2006; Monson et al., 2012). Crucially, recent evidence supports the view that harmonic vowel resonance and formant-harmonic alignment transcend purely physiological or acoustic phenomena, proving to be deeply relevant for auditory integration and neural streaming in singing (Saus et al., 2025). Furthermore, research indicates that emotional states modulate these high-frequency components; specifically, anger is characterised by a deliberate increase in spectral energy within the *F*_3_–*F*_4_ range to project greater emotional intensity (Hakanpää et al., 2014, 2019, 2023a, 2023b; Sundberg, 2021).

As noted above, this study primarily focuses on the expression of emotion in musical performance. We specifically consider the acoustic parameters most closely linked to emotional expression in singing. While numerous emotions are frequently present in classical music, their interpretation and expression pose a significant challenge for most singers. Various studies indicate that basic emotions such as joy, anger, and sadness are the most readily identifiable in music, whereas other emotions are generally more difficult to discern; this may be due to the ambiguity of the eliciting stimulus (Akkermans et al., 2019; De Gelder and Vroomen, 1996; Gabrielsson, 2001; Gabrielsson and Lindström, 2001; Juslin, 1997). Crucially, evaluating emotional expression in singing requires integrating the performer’s internal affective experience with the objective measurement of their acoustic markers. Grounded in the component process model of emotion, a genuine felt emotion triggers involuntary neurovegetative and somatic responses (Johnstone and Scherer, 2000; Scherer, 2009). These induced affective states alter subglottic pressure, laryngeal muscle tension, and the geometric configuration of the vocal tract articulators (Klasen et al., 2013; Sundberg, 1974). This underlying biomechanical restructuring drives the subsequent systematic modulations in the acoustic architecture of the voice, ensuring that the analysed acoustic markers reflect real physiological processing rather than mere conscious, technical simulation (Juslin and Laukka, 2003).

Extensive research (Juslin and Laukka, 2003; Patel et al., 2011; Scherer et al., 2017) has investigated how emotional expression induces systematic modulations in acoustic pattern relative to neutral vocal baselines. Specifically, Scherer et al. (2017) identified discrete acoustic patterns within the singing voice, noting substantial similarities between sadness and tenderness, and between joy and pride. Low-arousal categories —sadness and tenderness— are typically characterised by diminished loudness and dynamics, alongside reduced high-frequency spectral energy. Notably, sadness is distinguished from tenderness by higher formant amplitudes. This acoustic pattern fundamentally contrasts with anger, which is defined by high arousal and power, whereas sadness and tenderness reflect a physiological absence of power and low activation (Scherer et al., 2017).

The current study investigates joy and anger (including rage) as they represent the two divergent branches of emotional activation described in the Vector Model of Affect (Bradley et al., 1992). These primary high-arousal, high-power emotions emanate from a common neutral baseline toward extremes of activation, yet are primarily differentiated by their affective valence. Research has delineated distinct acoustic patterns that distinguish these categories (Patel et al., 2011; Scherer et al., 2017), suggesting that valence acts as a modulating factor even when activation levels are shared. Although both emotions exhibit high intensity and marked dynamic variability, joy is characterized by a substantial dynamic range indicative of energy and enthusiasm, whereas anger presents more abrupt amplitude peaks that evoke an explosive quality. Therefore, focusing on these diametrically opposed states allows for a precise examination of how positive and negative valences influence the acoustic signal at maximum levels of energetic activation. Regarding frequency, both states involve an elevated F_0_; however, joy exhibits considerable pitch variability (liveliness), while the more restricted variability in anger imparts a tighter, more focused vocal quality. Vibrato further differentiates the two: joy typically manifests as fast and broad, contributing to a sense of lightness, whereas anger is associated with rapid, irregular oscillations reflecting physiological agitation and a lack of emotional control. Finally, while both emotions demonstrated high energy in the upper formants (*F*_3_, *F*_4_, and *F*_5_), the specific configuration of these resonances determined the resulting vocal quality. In states of anger, a more concentrated clustering between *F*_3_ and *F*_4_ was observed, which produced a more “vibrant” and “expansive” timbre along with a greater professional projection; this resulted in the characteristic penetrating and “pressed” quality due to the increased glottal adduction and heightened subglottic pressure driven by the physiological arousal of the emotion, which induces a constriction of the vocal tract rather than resonance optimisation. Conversely, a greater convergence between F_3_ and *F*_5_ was associated with the presence of “squillo” and optimal brilliance, particularly during states of genuine joy, which resulted in a bright and resonant timbre (Scherer et al., 2017).

A critical methodological consideration in this field is that the emotional expression in lieder is intrinsically bound to the prescriptive lyrical and musical content of the piece. Consequently, in this study, purely vocal factors are not isolated from the semantic and structural meaning of the underlying repertoire. Instead, the research focuses on the modulation of felt emotions—as opposed to merely perceived emotions (Evans and Schubert, 2008)—under the premise that affective induction is most observable when using ecologically validated stimuli (Douglas-Cowie et al., 2007; Bänziger et al., 2012). This induction effect is further amplified by emotional congruence, where the induced state aligns with the valence of the musical fragment (Wendrich et al., 2010). Furthermore, this investigation deliberately excludes aesthetic emotions related to the perception of beauty or form (Cochrane et al., 2013), focusing strictly on the psychophysiological response to the primary induced affect. By prioritising felt emotion within a congruent framework, the study aims to capture the authentic physiological shifts that govern the professional singing voice. This approach is fundamentally aligned with the 4E/5E paradigm of cognitive science—a framework where Giovanna Colombetti has been a pivotal figure in defining emotive and affective cognition (Colombetti et al., 2018)—which posits that affective capacities are embodied (realised by physiology and agency) and enacted (reflective of first-person activities of sense-making and meaning-creation). Within this framework, the engagement with ecologically validated resources serves as a form of ‘scaffolding’—external structures that generate real-time feedback loops to transform the performer’s affective profile (Colombetti and Krueger, 2015). By facilitating the construction of an ‘affective niche’—defined as a self-styled environment where affective states take shape and thrive (Colombetti, 2014; Colombetti and Roberts, 2015; Krueger, 2014; Krueger, 2014)—the research examines how the singer’s ‘emotional mind’ transcends a mere technical representation. Instead, it embodies the affect, driving an authentic biomechanical restructuring of the vocal tract—characterised by shifts in subglottic pressure and laryngeal muscle tension—which ensures the validity of the resulting acoustic markers (Johnstone and Scherer, 2000; Scherer, 2009).

To conclude, whilst lyrical technique necessitates rigorous control over vocal production, current literature suggests that emotional arousal can systematically reconfigure an interpreter’s acoustic patterns. The inherent complexity of this interaction —in which artistic intent must be balanced against affective authenticity— demands a multi-parametric analysis addressing both discrete acoustic parameters and their intrinsic relationships. Accordingly, this study aims to analyse the extent to which induced emotion anger/rage as well joy modulates the acoustic parameters of the singing voice in professional lyrical singers under their respective conditions of affective congruence and incongruence. Through quantitative and qualitative analyses, the impact of these states on vocal source dynamics will be evaluated by examining the *F*_0_, SPL, and vibrato production. Furthermore, vocal filter strategies will be examined, including the configuration of formants (*F*_1_–*F*_4_), the “*R*_1_:*f*_0_ tuning’ phenomenon, and the formation of the SFC as resonance optimisation mechanisms. Finally, this operationalisation will be contrasted with the participants’ felt emotional experience, validated by the PANAS and BDI-II scales, to integrate the acoustic response with affective authenticity in professional performance.

## Methods

### Participants

Three professional soprano singers participated in the study with an average age of 42.27 years (SD = 0.86). Following the taxonomy of singers used as subjects in scientific research, all participants were classified as Category 2.1: International Opera Principals (Bunch and Chapman, 2000). All subjects had completed their tertiary studies at a Higher Conservatory of Music, which aligns with the training requirements for this category, and they maintain active professional careers performing in operas and lyric theatre (zarzuela) at both national and international levels. One of the inclusion criteria was no history of vocal pathology in the past year. No participating singer presented emotional disorders that could affect the study’s outcomes. Therefore, the singers completed the Beck Depression Inventory (BDI) twice with a time interval of 15 days, with the aim of establishing the absence of emotional disorders.

### Materials

#### Musical excerpts

The study utilised excerpts from established musical works, specifically known Lieder. The repertoire master analysed the musical, textual, contextual, and creative elements of the songs to ensure they aligned with the parameters of the target emotions. The three singers maintained close collaboration with the repertoire master to select and adapt the musical excerpts for performance. The song chosen to represent Joy was ‘*El tralalá y el punteado*’, while ‘*La maja dolorosa*’ was selected for Anger/Rage; both feature music by Enrique Granados and texts by Fernando Periquet.

The repertoire master identified that, in these specific works, both emotions reach their resolution at the climax of the musical phrase, specifically on the words ‘cantando’ (for joy) and ‘muerto’ (for anger). For the acoustic analysis, the focus was placed on the syllable ‘do’ (E_5_, 659.26 Hz) in the joy excerpt and the syllable ‘to’ (B_4_, 493.88 Hz) in the anger excerpt.

It is significant that ‘*La maja dolorosa*’ was employed for the Anger/Rage condition because the excerpt explores the profound concept of pain in the face of death. Granados himself distinguished between three distinct manifestations of pain within this work: the immediate shock following death, the pain expressed through tears, and the enduring sorrow of the time thereafter. This thematic depth provided the necessary framework for the singers to elaborate the meaning of the text and achieve the required emotional induction.

Once the musical excerpts were selected, the pianist recorded the accompaniments so that the singers could study them in advance using a consistent baseline for their subsequent recordings. This approach ensured a degree of homogeneity regarding the performance tempo, thereby facilitating the comparison between different singers and experimental conditions.

#### Instrumentation or individual difference measures

The Spanish version of the Positive and Negative Affect Schedule (PANAS; Watson and Clark, 1994; Sandin et al., 1999) is a questionnaire used to measure general emotional state. The questionnaire includes 20-items with 10 negative and 10 positive affects. This study employed three distinct dimensions, as put forth by Watson and Clark (1994). The first dimension is characterized by Positive Affect items, specifically including ‘enthusiastic,’ ‘happy,’ ‘joyful,’ ‘interested,’ and ‘determined.’ The second dimension, labelled Hostile, incorporates items such as ‘hostile,’ ‘scornful,’ ‘disgusted,’ ‘loathing,’ and ‘angry.’ Lastly, the third dimension, Tense, consists of items like ‘scared,’ ‘afraid,’ ‘frightened,’ and ‘shaky’.

The Spanish version of the Beck Depression Inventory II (BDI-II; Beck et al., 1996; Sanz et al., 2011) is a self-report of 21 items that assesses the existence and severity of symptoms of depression.

#### Recording and acoustic analyses of the singing samples

Acoustic voice analysis is a non-invasive tool that provides objective data on vocal production. However, its interpretation requires established reference norms, and its reliability is contingent upon factors such as microphone type, ambient noise, analysis software, and the specific acoustic parameters employed, as these variables can significantly impact results (Titze and Winholtz, 1993). To ensure optimum recording quality, Barsties and De Bodt (2015) recommend the use of: (1) condenser microphones, preferably head-mounted; (2) cardioid polar patterns; (3) a frequency range of 20–20,000 Hz; (4) a flat frequency response with a maximum deviation of 2 dB; (5) an equivalent noise level of ≤ 25 dB; (6) a maximum sound pressure level of ≥ 126 dB SPL for a 3% total harmonic distortion; and (7) high sensitivity, with a minimum of −60 dB. Accordingly, the recordings for this study were conducted in a professional recording studio, where all necessary technical conditions for the accurate registration of the singing voice were met.

Recordings were edited using Adobe Audition CS6. To ensure acoustic consistency while preserving the expressive dynamic range, vocal intensity was peak-normalised at the subject level. This process was professionally executed by the recording studio’s sound engineers responsible for the sessions, rather than through automated acoustic software such as Praat. Specifically, for each vocalist, a single gain factor was calculated based on their highest-intensity recording and subsequently applied to all their samples. This expert engineering approach ensured that relative acoustic intensity variability across the emotions was strictly retained and not artificially levelled, thus preserving the psychoacoustic integrity of the performances for subsequent analysis. Recordings were analyzed with Praat (Boersma and Weenink, 2013), a specialized software tool widely used in phonetics and speech-language pathology for high-precision acoustic analysis. This software allows for the identification and measurement of different vocal parameters—such as *F*_0_, intensity, harmonics, and noise—which are essential for characterizing emotional expression in the singing voice.

Acoustic analysis was performed using Praat via a customised protocol designed to prevent estimation errors driven by the wide harmonic spacing typical of soprano singing. The spectrogram window length was set to 0.05 s (50 ms) to yield a narrowband display capable of isolating individual harmonics. The maximum tracking range was elevated to 6,000 Hz (Burg algorithm, 5 formants) to capture the severe upward shifts of *F*_1_ and *F*_2_, and to define the Singer’s Formant Cluster (*F*_3_–*F*_5_) between 2,500 and 4,500 Hz. Formant tracks were manually cross-verified against the spectrogram, adjusting algorithm parameters adaptively if harmonic tracking occurred. To neutralise vibrato-driven laryngeal modulation, extraction was strictly confined to the stable portion of the vowel /o/ using a time-averaged protocol across multiple complete cycles, discarding static time-points. Resonance tuning (*R*_1_:*f*_0_ tuning) was operationally identified by the mathematical convergence in hertz between the extracted *F*_1_ and the fundamental frequency (*f*_0_). Finally, intensity levels were externally calibrated prior to the recording sessions using a reference sound pressure level meter positioned at the exact recording distance. This established the mathematical conversion factor between relative digital gain (dB FS) and absolute room acoustic pressure (dB SPL), ensuring that the reported maximum intensity (*I*__max_) metrics represent physically real sound pressure levels.

The selection process for the acoustic samples followed a hierarchical approach, beginning with the identification of the musical phrases that best represented the emotional and semantic climax of each excerpt. For the ‘Joy’ condition, the phrase “*Pues hay cosas que yo, siempre cantando*” was selected, while for ‘Anger/Rage’, the analysis focused on “*No, no, tú no has muerto*.” Within these phrases, the target words *“can-tan-do”* and “*muer-to*” were identified as the primary vehicles for emotional expression. Specifically, the analysis was narrowed down to their final syllables, *“do”* and *“to”* respectively, as these word-final positions capture the stabilized emotional trajectory at the conclusion of the musical gesture. To ensure phonetic consistency, the vowel /o/ was maintained as the common analytical unit across both conditions. Regarding the musical and acoustic parameters, the syllable *“do”* in the ‘Joy’ excerpt is a quarter note on E_5_ (659.26 Hz), slurred from the preceding syllable *“tan”* (eighth notes G_4_ and E_4_). In contrast, the syllable” *to”* in the ‘Anger/Rage’ excerpt corresponds to a B_4_ (493.88 Hz), consisting of a quarter note slurred to an eighth note and preceded by an eighth note C_5_ on the syllable *“muer”*. Despite the differences in tessitura—necessitated by the use of ecologically validated repertoire—the reliability of the spectral measurements was ensured through the extraction of stable tracks. These partitions, ranging from 1.0 to 2.0 s, provided a representative sample of steady-state phonation at the end of the expiratory cycle, where the laryngeal-pharyngeal configuration is most consistent.

### Emotional induction program

The emotional induction programme was explicitly designed to evaluate and compare the impact of affective congruency versus incongruency on the vocal performance of three professional singers. This methodological approach enables a precise isolation of the manner in which involuntary physiological activations alter technical instrumental execution when the artistic intent of the repertoire and the performer’s actual emotional state either mutually reinforce one another (congruent condition) or enter into direct conflict (incongruent condition).

To experimentally manipulate these affective states, various cinematographic fragments were selected from the validated FilmStim database (Schaefer et al., 2010), applying strict criteria of psychometric equivalence across their dimensions of arousal and valence.

For the induction of the sad/negative state (Anger/Rage), two highly distressing clips were selected from the same cinematographic work, thereby ensuring homogeneity in both the visual aesthetics and the narrative context of the stimuli. The first of these belongs to *Schindler’s List* [1]—a scene depicting the transport of lifeless bodies—which registers an *Arousal* score of 5.88 and a *Negative Affect* score of 2.62. The second fragment corresponds to *Schindler’s List* [2]—a sequence in which the camp commander randomly shoots prisoners from his balcony—a stimulus that offers an excellent metric equivalence by registering a *Negative Affect* score of 2.43 and an *Arousal* score of 5.19, whilst additionally positioning itself as the most effective stimulus within the database for the specific induction of anger.

Conversely, for the induction of a joyful mood (positive affect), two high-energy comic clips were deployed to ensure an equivalent activation profile. On the one hand, the fake orgasm scene from When Harry Met Sally was chosen, which is classified with a Positive Affect score of 2.32 and a moderate-to-high Arousal score of 4.71. On the other hand, the chaotic television game show sequence from the film “*Les trois frères*” was selected, exhibiting an almost identical psychometric performance, with a Positive Affect index of 2.32 and an Arousal score of 4.70.

### Experimental conditions

The study established two primary conditions based on the emotional valence of the musical excerpts (i.e., Anger/Rage and Joy) and three sequential levels of emotional induction (i.e., Non-induction baseline, Congruent induction, and Incongruent induction). For each musical excerpt, the protocol strictly commenced with the Non-induction condition, which served as a control baseline where participants performed the repertoire without any external affective stimulation. This was sequentially followed by the Congruent condition, in which an affective state was induced that precisely aligned with the emotional valence of the music (e.g., inducing anger before a piece about rage) through the presentation of validated film fragments (e.g., *Schindler’s List* for anger). Finally, the trial concluded with the Incongruent condition, defined as a state of deliberate affective dissonance where the induced emotion directly conflicted with the musical excerpt (e.g., inducing amusement/joy before a piece about anger and rage).

The decision to implement this specific, unvarying sequence—moving invariably from non-induction to congruent, and finally to incongruent conditions—was theoretically driven by the premise that performing an emotional musical excerpt inherently elicits a specific affective state within the singer. Consequently, the emotional effect of the repertoire was expected to be intensified during the congruent induction phase, whilst the inclusion of the incongruent induction served to facilitate the detection of marked technical and acoustic differences relative to the first two states. To eliminate any potential confounding variables or carryover effects arising from this fixed sequential architecture, a rigorous control protocol was applied: the order of presentation of the musical pieces within each block was determined in a fully randomised manner (Anger/Rage versus Joy) for all participants.

Furthermore, to neutralise the possibility of any single film clip being disproportionately effective or triggering an idiosyncratic response in a particular participant, a meticulous counterbalancing and rotation protocol was enforced. The specific cinematic stimuli assigned to each trial were systematically rotated and balanced across the experimental sessions among the three sopranos. By ensuring that every film fragment was equally distributed across the tasks, any potential variance in individual emotional reactivity was statistically controlled and neutralised, thereby safeguarding the methodological rigor and internal validity of the experiment.

### Procedure

The studies involving human participants were reviewed and approved by the Ethics Committee of the University of Salamanca (Comité de Ética de la Investigación de la Universidad de Salamanca). The studies were conducted in accordance with the local legislation and institutional requirements. The participants provided their written informed consent to participate in this study. The singers’ professional services were formally contracted, and they received financial compensation for their participation. At the beginning of the process, participants were informed of the study’s aims and their rights. Subsequently, they completed the BDI-II questionnaire to evaluate their emotional state at two different times within a two-week interval. The singers had meetings with the repertoire pianist, with the aim of choosing those lieder songs that best suited their voices and represented the indicated emotions.

In the second phase, experimental tests were conducted in a professional recording studio during morning sessions. The process began by establishing two stable emotional references: participants completed the initial PANAS questionnaire (Baseline 1) and, after a strict 20-min rest interval, completed it a second time (Baseline 2). Subsequently, initial sound adjustments and a vocal warm-up were performed, during which recording levels were calibrated based on each vocalist’s loudest execution to prevent clipping.

To mitigate order biases, the presentation order of the two musical repertoire blocks (Joyful and Anger/Rage) was randomized for each participant, implementing a structural 60-min rest period between the two blocks to ensure complete vocal and emotional recovery. Within each musical block, the tasks followed a rigid sequence across all participants to ensure a standardized progression: No-Induction, Congruent, and Non-Congruent. For the No-Induction baseline task, participants performed the assigned musical excerpt without any prior emotional induction. For the subsequent emotional induction conditions, the process was conducted individually in a dimly lit room where participants had to be fully relaxed before viewing selected movie fragments. Immediately after watching the assigned clips, without engaging in any other activity, they proceeded to interpret the corresponding musical excerpt.

Crucially, across the induction conditions, the PANAS questionnaire was systematically administered at two specific moments: immediately before the induction condition (Pre-test) and immediately after the induction condition (Post-test). This Post-test assessment served as an immediate manipulation check to verify the effectiveness of the film clips; directly subsequent to this, and without engaging in any other activity, participants proceeded to the vocal performance of the musical excerpt. Strict 20-min recovery intervals were implemented between these three conditions to eliminate physical fatigue and prevent any lingering emotional contamination from the previous trial.

## Results

In accordance with the research objectives established for this study, the following results are presented through a multi-layered analytical approach structured by musical excerpt. Crucially, to prevent acoustic confounds derived from pitch height and tessitura, each musical piece is analyzed as an independent experimental block, entirely avoiding cross-repertoire comparisons. First, a detailed individual analysis is conducted for each singer, integrating both self-reported emotional states and vocal acoustic parameters across the congruent and incongruent conditions. This case-by-case examination categorises the vocal architecture into vocal source dynamics (SPL, *F*_0_, and vibrato) and vocal filter and resonant strategies (*F*_1_–*F*_5_ and the SFC, LTAS mean, Alpha Ratio and “*R*_1_:*f*₀ tuning), allowing for a precise identification of how induced affect reconfigures each participant’s unique physiological output. Second, a Global Interpretation is provided for each excerpt, establishing comparative relationships between the singers within that specific musical context to identify common patterns or divergent strategies in both emotional variables and acoustic modifications. This dual-level structure—moving from individual performance to collective synthesis—provides a comprehensive overview of the mechanisms underpinning affective expression in professional lyrical singing, processed first as an isolated block for the ‘Anger/Rage’ excerpt and subsequently as an independent block for the ‘Joy’ excerpt.

### Anger/rage musical excerpt

A comparison between conditions revealed that Singer 1 maintained a notably stable affective profile during the Anger/Rage excerpt. While her scores remained virtually unchanged during the non-induction condition, the congruent condition triggered subtle but directed shifts: her Positive affect decreased by 0.50 points (from 2.70 pre-test to 2.20 post-test), while both her Tension and Hostility scores increased by 0.33 points (Table 1). This reflects a controlled affective distance, where the singer’s physiological output was dictated by the excerpt’s technical requirements rather than by a marked subjective emotional shift.

**Table 1:** Mean (and standard deviation) in the PANAS dimension of the singers under different induction conditions by musical excerpts.

			Base line		Non-induction			Congruent			Incongruent			M (*SD*)
			a	b	Pre	Post	Δ	Pre	Post	Δ	Pre	Post	Δ	
Anger/Rage	Singer 1	Positive	3.00	2.90	2.50	2.60	0.1	2.70	2.20	−0.5	2.80	2.90	0.1	2.70 (0.26)
		Tense	1.00	1.17	1.17	1.00	−0.17	1.00	1.33	0.33	1.33	1.33	0	1.17 (0.15)
		Hostility	1.00	1.00	1.00	1.00	0	1.00	1.33	0.33	1.00	1.00	0	1.04 (0.12)
	Singer 2	Positive	3.10	3.00	2.60	2.50	−0.1	2.80	1.00	−1.8	2.60	3.30	0.7	2.61 (0.71)
		Tense	1.17	1.33	1.33	1.17	−0.16	1.33	3.67	2.34	1.33	1.17	−0.16	1.58 (0.85)
		Hostility	1.00	1.00	1.00	1.00	0	1.00	3.67	2.67	1.17	1.00	−0.17	1.36 (0.94)
	Singer 3	Positive	2.20	1.70	1.90	1.90	0	1.90	1.00	−0.9	1.90	1.70	−0.2	1.78 (0.35)
		Tense	1.83	2.00	2.17	1.83	−0.34	2.00	4.00	2	2.17	2.00	−0.17	2.25 (0.72)
		Hostility	1.33	1.33	1.33	1.00	−0.33	1.00	4.83	3.83	1.33	1.00	−0.33	1.64 (1.30)
Joy	Singer 1	Positive	3.00	2.50	2.70	2.80	0.1	2.60	3.60	1	3.00	2.70	−0.3	2.86 (0.35)
		Tense	1.00	1.17	1.00	1.00	0	1.00	1.00	0	1.00	1.17	0.17	1.04 (0.08)
		Hostility	1.00	1.00	1.00	1.00	0	1.00	1.00	0	1.00	1.00	0	1.00 (0.00)
	Singer 2	Positive	3.10	2.60	2.90	2.70	−0.2	2.80	2.60	−0.2	2.50	2.60	0.1	2.73 (0.20)
		Tense	1.17	1.33	1.33	1.17	−0.16	1.33	1.00	−0.33	1.33	1.17	−0.16	1.23 (0.12)
		Hostility	1.00	1.00	1.00	1.00	0	1.00	1.00	0	1.00	1.33	0.33	1.04 (0.12)
	Singer 3	Positive	2.20	1.90	2.10	2.00	−0.1	1.90	2.00	0.1	2.00	1.70	−0.3	1.98 (0.15)
		Tense	1.83	2.17	2.17	1.83	−0.34	1.83	1.67	−0.16	1.1.67	1.83	0.66	1.90 (0.19)
		Hostility	1.33	1.33	1.33	1.33	0	1.50	1.33	−0.17	1.33	1.67	0.34	1.39 (0.13)

El valor Δ se calcula como Post–Pre.

Regarding her vocal source dynamics (Table 2), Singer 1 achieved a peak intensity (*I_-max_*) of 72.80 dB and a maximum fundamental frequency (*F*_0-max_) of 551.89 Hz. Her primary expressive resource remained a subtle modulation of vibrato morphology, which featured a Rate of 5.15 Hz and an Extent of 74.03 cents. Although her frequency stability remained relatively high, qualitative observations indicated that her vibrato became more “pointed” or “accentuated” to represent the negative affect, reflecting a controlled increase in glottal tension.

**Table 2:** Mean (and standard deviation) of glottal source dynamics (intensity, frequency, and vibrato) of the singers under different induction conditions of the anger/rage excerpt.

		Conditions			M (*SD*)
		Non-induction	Congruent	Incongruent	
Singer 1	*I*_-max_ (dB)	69.25	72.80	67.78	69.94 (2.58)
	*F*_0-max_ (Hz)	561.83	551.89	554.88	556.20 (5.10)
	Vib. extent (cents)	78.68	74.03	59.23	70.65 (10.16)
	Vib. rate (Hz)	5.28	5.15	5.27	5.23 (0.07)
Singer 2	*I*_-max_ (dB)	63.41	70.39	64.89	66.23 (3.68)
	*F*_0-max_ (Hz)	541.97	566.69	542.11	550.26 (14.23)
	Vib. extent (cents)	53.36	65.51	56.34	58.40 (6.33)
	Vib. rate (Hz)	5.76	5.46	5.48	5.57 (0.17)
Singer 3	*I*_-max_ (dB)	70.00	70.90	68.22	69.71 (1.36)
	*F*_0-max_ (Hz)	528.20	550.12	539.29	539.20 (10.96)
	Vib. extent (cents)	104.31	116.80	127.82	116.31 (11.76)
	Vib. rate (Hz)	5.16	5.02	4.87	5.02 (0.15)

These source adjustments were synergistically accompanied by specific configurations in her vocal filter and resonance markers (Table 3). Her glottal source exhibited a clear shift towards “pressed” phonation, with the *H*_1_–*H*_2_ ratio dropping from a baseline of 4.3 dB to a negative value of −6.1 dB, providing physical evidence of increased medial compression and laryngeal tension. While she did not rely on extreme SFC amplification (recording an intensity of −74.33 dB), her LTAS mean increased slightly to 7.69 dB, and she maintained an Alpha Ratio of −22.87 dB. Furthermore, the consistent absence of “*R*_1_:*f*_0_ tuning” was confirmed by a pronounced divergence of 110.72 Hz, as her resonance strategy prioritised physiological constriction over the acoustic optimisation typical of positive affect. Ultimately, these markers confirm that the task induced a quantifiable vocal strain and spectral reconfiguration, even in the absence of a high-arousal emotional report.

**Table 3:** Mean (and standard deviation) of vocal filter and resonance markers (formants, SFC, and H1–H2 ratio, LTAS, Alfa ratio) of singers under different induction conditions for the anger/rage excerpt.

		Conditions			
		Non-induction	Congruent	Incongruent	M (SD)
Singer 1	*F*_1_ (Hz)	582.34	610.62	574.82	589.26 (18.88)
	*F*_2_ (Hz)	1202.04	1217.44	1149.45	1189.64 (35.65)
	*F*_3_ (Hz)	2848.61	2890.37	2857.86	2865.61 (21.93)
	*F*_4_ (Hz)	3430.52	3462.99	3397.82	3430.44 (32.59)
	*F*_5_ (Hz)	4595.15	4670.83	4510.11	4592.03 (80.41)
	*R*_1_:*f*_0_ tuning	74.16	110.72	68.47	84.45 (22.93)
	SFC (dB)	−51.00	−74.33	−76.39	−67.24 (14.12)
	Ratio H1–H2 (dB)	4.3	−6.1	23.3	7.17 (14.91)
	LTAS mean (dB)	6.94	7.69	6.70	7.11 (0.52)
	Alfa ratio (dB)	−10.15	−22.87	−25.17	−19.40 (8.09)
Singer 2	*F*_1_ (Hz)	647.56	589.52	672.64	636.57 (42.64)
	*F*_2_ (Hz)	1032.78	1051.13	1096.59	1060.17 (32.85)
	*F*_3_ (Hz)	2845.04	2802.11	2949.8	2865.65 (75.97)
	*F*_4_ (Hz)	3708.36	3610.84	3616.11	3645.10 (54.85)
	*F*_5_ (Hz)	4560.24	4620.30	4700.12	4600.00 (52.20)
	*R*_1_:*f*_0_ tuning	140.48	77.85	168.22	128.85 (46.29)
	SFC (dB)	−89.48	−83.63	−78.00	−83.70 (5.74)
	Ratio H1–H2 (dB)	−17.8	−0.01	−7.2	−8.34 (8.95)
	LTAS mean (dB)	1.15	2.42	3.68	2.42 (1.27)
	Alfa ratio (dB)	−28.14	−21.56	−17.03	−22.24 (5.59)
Singer 3	*F*_1_ (Hz)	512.76	525.89	523.76	520.80 (7.05)
	*F*_2_ (Hz)	1063.71	1126.14	1033.63	1074.49 (47.19)
	*F*_3_ (Hz)	2663.60	2652.83	2612.95	2643.13 (26.68)
	*F*_4_ (Hz)	3633.60	3623.73	3587.45	3614.93 (24.30)
	*F*_5_ (Hz)	4615.16	4650.21	4560.10	4608.49 (45.42)
	*R*_1_:*f*_0_ tuning	45.33	−52.97	61.14	17.83 (61.82)
	SFC (dB)	−81.84	−79.09	−87.75	−82.89 (4.42)
	Ratio H1–H2 (dB)	20.1	−7.3	7.1	6.63 (13.70)
	LTAS mean (dB)	7.32	7.32	5.70	6.78 (0.94)
	Alfa ratio (dB)	−22.40	−24.70	−25.66	−24.25 (1.68)

In the case of the incongruent condition, Singer 1 faced a distinct state of affective dissonance, where the induction of genuine joy—which caused her Positive affect score to increase by only 0.10 points from a high pre-test score of 2.80 to post-performance score of 2.90 (Table 1)—conflicted with the technical demands of the anger performance. Quantitatively, this emotional misalignment triggered a generalised disorganisation of phonation, suggesting that the internal conflict between the induced emotion and the performed task compromised the fine motor coordination required for stable professional singing.

Her vocal source dynamics (Table 2) reflected this instability; her peak intensity (*I*_max_) decreased to 67.78 dB and her *F*_0-max_ was recorded at 554.88 Hz. This state was further manifested through a severe degradation of vibrato regularity and a reduction in vibrato extent to 59.23 cents, providing physical evidence of a marked loss of glottal control under affective dissonance.

Complementing these source disruptions, a parallel collapse of resonant structures was observed in her vocal filter and resonance markers (Table 3). Her glottal source shifted from the ‘pressed’ quality of the congruent state to an excessively lax emission, with the *H*_1_–*H*_2_ ratio reaching its peak of 23.3 dB. This technical decline was further confirmed by the *F*_5_ dropping to its minimum value of 4510.11 Hz and the Alpha Ratio falling to its lowest point of −25.17 dB. Ultimately, these markers, along with a decrease in LTAS Mean to 6.70 dB, demonstrate that the divergence between induced affect and artistic intent undermines the spectral robustness required for stable professional vocal projection.

Singer 2, under the congruent condition of the Anger/Rage excerpt, exhibited a strategic acoustic reconfiguration to intensify the expression of anger, triggered by a marked psychological shift where both Hostility and Tension scores reached an identical peak of 3.67; however, the magnitude of the increase was greater for Hostility, which rose by 2.67 points (from a pre-test level of 1.00), than for Tension, which increased by 2.34 points (from a pre-test level of 1.33) (Table 1). This state of high arousal indicated that her vocal modulations were the result of a profound affective engagement coupled with professional technical regulation.

An examination of the vocal source dynamics (Table 2) showed that the participant achieved a peak intensity (*I_max_*) of 70.39 dB and *F*_0-max_ of 566.69 Hz. Emotional intensity was primarily conveyed through an expanded yet stable vibrato morphology, featuring an extent of 65.51 cents and a rate of 5.46 Hz. These parameters provided the necessary dramatic energy for the Anger excerpt without compromising periodic stability, representing a controlled channelling of aggression into the vocal signal.

These source dynamics were synergistically supported by her vocal filter and resonance markers (Table 3). Her glottal source reached an *H*_1_–*H*_2_ ratio of −0.01 dB, reflecting an energised and balanced phonatory effort compared to her naturally ‘pressed’ baseline. This resonant strategy was further characterised by enhanced vocal brilliance, evidenced by an intensified SFC (−83.63 dB) and supported by her upper formant positioning at 2802.11 Hz (*F*_3_), 3610.84 Hz (*F*_4_), and 4620.30 Hz (*F*_5_). Ultimately, these adjustments resulted in a robust LTAS Mean of 2.42 dB and an improved Alpha Ratio of −21.56 dB, providing physical evidence of the spectral energy concentration required to project the penetrating quality of anger.

Turning to the incongruent condition for the Anger/Rage excerpt, Singer 2’s response was characterised by a resilient but strained technical strategy. Despite the emotional dissonance, her Positive affect score increased by 0.70 points, while her Tension and Hostility scores decreased by 0.16 and 0.17 points, respectively (Table 1). This psychological shift forced her to reinforce her projection mechanisms to compensate for the resulting conflict between the induced emotion and the task demands.

Her vocal source dynamics (Table 2) reflected an energetic effort that was decoupled from motor stability; she recorded a peak intensity (*I_max_*) of 64.89 dB and a *F*_0-max_ of 542.11 Hz. Spectrographic analysis revealed a pronounced degradation of vibrato, with her extent falling markedly to 56.34 cents (at a rate of 5.48 Hz) from the 65.51 cents achieved during the congruent condition. This reduction provided physical evidence of the loss of glottal control and fine motor coordination under affective misalignment.

Beyond the vocal source, her resonance markers and vocal filter (Table 3) showed specific configurations aimed at maximising spectral brilliance, even at the expense of phonatory ease. This was evidenced by her SFC (2500–3,500 Hz) reaching −78.00 dB and her Alpha Ratio improving to its most robust value of −17.03 dB, surpassing both her baseline and congruent states in spectral brilliance. However, her glottal source showed signs of strain with an *H*_1_–*H*_2_ ratio of −7.2 dB, indicating a return to a more ‘pressed’ and constricted phonation compared to the balanced −0.01 dB of the congruent condition. Ultimately, despite maintaining a high LTAS Mean of 3.68 dB, the inconsistent vibrato and increased glottal pressure resulted in a ‘veiled’ or ‘harsh’ vocal quality, proving that emotional misalignment compromises harmonic clarity even when resonance remains high.

Finally, Singer 3, under the congruent condition for the Anger/Rage excerpt, demonstrated a highly efficient technical response supported by the most intense psychological reaction among the participants. Her Hostility score rose from a pre-test level of 1.00 to 4.83, representing an increase of 3.83 points from its pre-test baseline of 1.00, while her Tension score reached 4.00, increasing by 2.00 points from a pre-test level of 2.00 (Table 1). Concurrently, her Positive affect score decreased by 0.90 points. This state of high arousal indicated that her vocal modulations were the result of profound affective engagement coupled with a forceful physiological strategy. Data derived from the vocal source dynamics (Table 2), revealed a peak intensity (*I*_max_) of 70.90 dB and a *F*_0-max_ of 550.12 Hz. Her primary vehicle for expressing the emotional ‘surge’ was the dramatic expansion of her vibrato morphology, which reached an extent of 116.80 cents at a rate of 5.02 Hz. This robust frequency modulation provided the necessary dramatic energy for the excerpt while ensuring a stable and professional emission.

In parallel with the source dynamics, the vocal filter and resonance markers (Table 3) exhibited configurations that provided synergistic support to the overall sound. Her glottal source shifted toward a ‘pressed’ phonation, evidenced by the *H*_1_–*H*_2_ ratio reaching −7.3 dB, which indicated a marked increase in medial compression and subglottic pressure. This powerful vocal constriction allowed her to channel the aggression of anger into a focused and ‘penetrating’ timbre without losing harmonic integrity. Quantitatively, this state was characterised by a robust LTAS mean of 7.32 dB and an Alpha Ratio of −24.70 dB. Furthermore, the persistent absence of “*R*_1_:*f*_0_ tuning” was confirmed by a pronounced divergence of −52.97 Hz, as her resonance strategy prioritised physiological constriction over the acoustic optimisation typical of joy. Her upper formant alignment, featuring an *F*_5_ at 4650.21 Hz and an SFC intensity of −79.09 dB, provided physical evidence of the high-frequency spectral energy required to achieve the necessary dramatic impact.

Regarding the incongruent condition for the Anger/Rage excerpt, Singer 3 exhibited a generalised phonatory disorganisation as the induction of joy conflicted with the task of performing anger. This state was characterised by an affective shift where her Positive affect score decreased by 0.20 points, while her Hostility score dropped by 0.33 points (Table 1). Furthermore, she started from a high baseline Tension level of 2.17 in the pre-test condition, which decreased by only 0.17 points to a post-performance score of 2.00 ( Δ = − 0.17), suggesting that the emotional misalignment undermined the psychophysiological readiness required for the task. In terms of vocal source dynamics (Table 2), the singer exhibited this instability through a peak intensity (*I*_max_) of 68.22 dB and a *F*_0-max_ of 539.29 Hz. The vibrato became markedly unstable, with the extent expanding to 127.82 cents and the rate falling to 4.87 Hz. This expansion in vibrato extent, coupled with the loss of periodicity, provided physical evidence of extreme vocal effort and a clear misalignment between emotional intent and fine motor control.

In tandem with these source disruptions, the vocal filter and resonance markers (Table 3) exhibited a systemic collapse of the resonant architecture. Her glottal source reflected this instability with an *H*_1_–*H*_2_ ratio of 7.1 dB, and a breakdown in resonance efficiency was evidenced by a “*R*_1_:*f*_0_ tuning” divergence of 61.14 Hz. Her upper formants reached their lowest points in this condition, with *F*_3_ at 2612.95 Hz, *F*_4_ at 3587.45 Hz, and *F*_5_ at 4560.10 Hz, causing the SFC intensity to weaken to −87.75 dB. Ultimately, these adjustments resulted in her minimum LTAS Mean of 5.70 dB and a drop in the Alpha Ratio to −25.66 dB, confirming that affective dissonance compromises the source–filter coupling and spectral robustness required for professional vocal projection.

### Global interpretation

The emotional induction for the Anger/Rage excerpt revealed a clear divergence in affective engagement among the participants (Table 1). While the protocol successfully elicited a pronounced increase in negative affect for Singers 2 and 3—characterised by marked peaks in Hostility and Tension—Singer 1 maintained high affective stability and psychological distance. This suggests that for Singers 2 and 3, the induction acted as a powerful internal catalyst for the aggressive vocal markers observed; whereas Singer 1 relied on a more detached, expert-controlled mechanism, allowing the musical requirements to dictate her physiological response rather than felt emotion. Notably, the incongruent phase triggered a unique conflict for Singer 1; the induction successfully elicited genuine joy, creating a direct affective interference with the technical demands of the anger performance. This unexpected shift challenged her habitual emotional regulation, potentially disrupting the fine coordination between the vocal source and filter by introducing a positive arousal state that was physiologically incompatible with the ‘penetrating’ and constricted quality required for the excerpt.

Regarding the vocal source dynamics (Table 2), all participants recorded their highest peak intensity levels (*I*_-max_) during the congruent induction, indicating a collective increase in vocal power as a response to the emotion of anger. Singer 2 exhibited the most pronounced physiological shift, with a + 6.98 dB increase from her baseline to 70.39 dB. However, the strategies employed to achieve this intensity varied markedly among participants. While Singers 2 and 3 demonstrated a marked upward shift in *F*_0-max_ (+24.72 Hz and +21.92 Hz, respectively) to support their increased volume, Singer 1 achieved her peak intensity (72.80 dB) despite a 9.94 Hz decrease in her *F*_0-max_ relative to the baseline. This divergence suggests that while Singers 2 and 3 relied on increased glottal tension and frequency to convey the high arousal of rage, Singer 1 maintained a more efficient phonatory balance, prioritising resonant reinforcement over laryngeal effort even under conditions of high-arousal negative affect.

Regarding vibrato production (Table 2), a divergent pattern emerged among the participants under the congruent condition. While vibrato rates remained remarkably stable across the group (ranging from 5.02 to 5.46 Hz), Singers 2 and 3 displayed a notable expansion in vibrato extent; Singer 2 increased by +12.15 cents (from 53.36 to 65.51 cents) and Singer 3 by +12.49 cents (from 104.31 to 116.80 cents). This broadening of the oscillation suggests an increase in subglottic pressure and a heightened emotional surge typical of high-arousal negative affect. Conversely, Singer 1 adopted a more restrained physiological strategy; rather than increasing frequency oscillations, she relied on subtle shifts in wave morphology—characterised by a more ‘pointed’ or ‘accentuated’ harmonic profile—to represent the aggressive quality of the affect. This suggests that while Singers 2 and 3 expressed the intensity of rage through mechanical expansion, Singer 1 prioritised glottal efficiency and spectral adjustments, maintaining a more controlled phonatory output even at peak intensity levels.

Simultaneously, the examination of vocal filter and resonant strategies (Table 3) determined how emotional induction modulated the configuration of formants and energy distribution. The acoustic configuration during the anger induction departed from resonance optimisation, as the modulation of anger favoured physiological constriction. Consistently, “*R*_1_:*f*_0_ tuning” was absent across all participants, with *f*_1_–*f*_0_ divergences exceeding 22.83 Hz. This lack of tuning, coupled with a marked widening of the inter-formant distance (*F*_1_–*F*_2_ gap) in Singers 2 and 3 (+76.39 Hz and +49.3 Hz, respectively), was observed alongside a reduction in spectral prominence. Despite increased formant separation between *F*_3_ and *F*_4_, all singers maintained spectral energy within the SFC region through heightened intensity and efficient excitation of higher resonances, contributing to a “penetrating” voice quality.

The impact of emotional incongruence triggered a generalised disorganisation of phonation and a pronounced reduction in technical stability across all three singers. This was manifested as a marked disruption of vibrato regularity, resulting in highly irregular and inconsistent patterns. Notably, in Singer 1 —who maintained high affective stability during the ineffective congruent induction— this disruption was specifically triggered by the successful elicitation of joy (Positive affect score of 2.90), demonstrating that affective dissonance, rather than technical vulnerability, undermined her professional baseline. Quantitatively, this state was evidenced by a reduction in her vibrato extent to 59.23 cents and an abrupt shift towards an excessively lax emission, with her *H*_1_–*H*_2_ ratio reaching a peak of 23.3 dB.

The most severe disruption occurred in Singer 3, whose wide and slow vibrato —characterised by a + 23.51 cent expansion in extent (reaching 127.82 cents) and a rate falling to 4.87 Hz— signified extreme vocal effort driven by forceful glottal compression and a complete misalignment between emotional intent and vocal production control. This physiological tension resulted in a systemic collapse of her resonant architecture, where her LTAS Mean fell to a minimum of 5.70 dB and her SFC intensity weakened to −87.75 dB. Collectively, these findings underscored that while professional singers employed effective strategies to project the ‘penetrating’ quality of anger, the presence of affective dissonance undermined the fine motor coordination and spectral robustness required for stable resonance.

Regarding the incongruent condition for the Anger/Rage excerpt, a substantial disruption of the previously observed physiological strategies emerged, with marked differences between the participants. While the congruent state fostered a ‘penetrating’ quality through controlled constriction, the incongruent induction triggered a loss of coordination between the vocal source and the filter. Singer 1 exhibited a notable collapse in her energy distribution, with her *F*_0-max_ falling markedly compared to her congruent performance, suggesting a lack of the subglottic pressure necessary to sustain the aggressive affect. In contrast, Singers 2 and 3 displayed a pattern of ‘hyper-constriction’ under incongruence; their vibrato became unstable and accelerated (reaching a rate of 5.72 Hz in Singer 3), and their *F*_1_–*F*_2_ gap narrowed, reflecting a state of vocal strain rather than expressive power. This divergence indicates that while Singer 1 responded to affective dissonance with a reduction in phonatory drive, Singers 2 and 3 reacted with increased laryngeal tension, highlighting that without emotional congruence, the technical execution of rage loses its resonance-based reinforcement and becomes physiologically costly.

### Joy musical excerpt

Regarding Singer 1, the emotional induction for the Joy excerpt proved to be uniquely effective. While her scores remained stable during the non-induction condition, the congruent condition triggered a marked emotional shift: her positive affect score rose by 1.00 point, increasing from a pre-test of 2.60 to a post-performance peak of 3.60 (Table 1). This value suggests that the congruent emotional state acted additively with the musical interpretation, fostering superior resonance optimisation and technical robustness. In this case, the ‘felt’ emotion reinforced the mechanical requirements of the score, creating a synergy that resulted in the observed acoustic enhancements.

She achieved her highest peak intensity (*I*_-max_) of 73.19 dB, with a controlled *F*_0-max_ of 742.75 Hz; this reflects a 21.82 Hz reduction compared to her 764.57 Hz baseline, suggesting a more efficient phonatory balance. This was complemented by a stable and sustained vibrato with an Extent of 132.87 cents, ensuring that the emotional buoyancy of joy was maintained with technical precision from the onset of phonation (Table 4). These source dynamics were synergistically supported by her resonance strategy, which featured an efficient “*R*_1_:*f*_0_ tuning” — with her F_1_ (726.10 Hz) precisely aligned with her *F*_0-max_ (742.75 Hz) and a notable expansion of her vowel space (F_1_–F_2_ gap) to 770.05 Hz (Table 5).

**Table 4:** Mean (and standard deviation) of glottal source dynamics (intensity, frequency, and vibrato) of the singers under different induction conditions of the joy excerpt.

		Conditions			M (SD)
		Non-induction	Congruent	Incongruent	
Singer 1	*I*_-max_ (dB)	72.70	73.19	70.97	72.29 (1.17)
	*F*_0-max_ (Hz)	764.57	742.75	734.37	747.23 (15.59)
	Vib. Extent (cents)	137.02	132.87	106.15	125.35 (16.75)
	Vib. Rate (Hz)	5.12	5.12	5.12	5.12 (0.00)
Singer 2	*I*_-max_ (dB)	72.28	75.10	75.29	74.22 (1.69)
	*F*_0-max_ (Hz)	733.39	731.71	758.61	741.24 (15.07)
	Vib. Extent (cents)	134.51	129.16	133.45	132.37 (2.83)
	Vib. Rate (Hz)	5.44	5.34	5.29	5.36 (0.08)
Singer 3	*I*_-max_ (dB)	78.78	77.46	79.82	78.69 (1.18)
	*F*_0-max_ (Hz)	729.71	715.78	728.54	724.68 (7.73)
	Vib. Extent (cents)	158.73	146.37	135.70	146.93 (11.53)
	Vib. Rate (Hz)	5.62	5.50	5.72	5.61 (0.11)

**Table 5:** Mean (and standard deviation) of vocal filter and resonance markers (formants, SFC, and H1–H2 ratio) of singers under different induction conditions for the joy excerpt.

		Conditions			M (SD)
		Non-induction	Congruent	Incongruent	
Singer 1	*F*_1_ (Hz)	712.17	726.10	673.22	703.83 (27.41)
	*F*_2_ (Hz)	1458.09	1496.15	1330.22	1428.15 (86.92)
	*F*_3_ (Hz)	2879.09	2829.24	2888.16	2865.50 (31.73)
	*F*_4_ (Hz)	3537.78	3531.94	3536.07	3535.26 (3.00)
	*F*_5_ (Hz)	4380.64	4620.61	4510.11	4503.79 (120.11)
	*R*_1_:*f*_0_ Tuning	11.61	14.96	15.98	14.18 (2.29)
	SFC (dB)	−64.17	−63.34	−71.82	−66.44 (4.67)
	Ratio H1-H2 (dB)	1.5	6.5	9.5	5.83 (4.04)
	LTAS Mean (dB)	15.25	17.65	12.68	15.19 (2.49)
	Alfa Ratio (dB)	−1.89	−6.09	−6.53	−4.84 (2.56)
				
Singer 2	*F*_1_ (Hz)	701.79	660.50	671.24	677.84 (21.42)
	*F*_2_ (Hz)	1408.56	1328.52	1351.08	1362.72 (41.27)
	*F*_3_ (Hz)	2761.29	2817.23	2818.04	2798.85 (32.53)
	*F*_4_ (Hz)	3661.43	3765.73	3640.81	3689.32 (66.97)
	*F*_5_ (Hz)	4385.20	4625.12	4480.07	4496.80 (120.83)
	*R*_1_:*f*_0_ Tuning	10.68	−13.13	−18.41	−6.95 (15.50)
	SFC (dB)	−78.85	−71.47	−71.06	−73.79 (4.39)
	Ratio H1-H2 (dB)	8.5	0	6.4	4.97 (4.43)
	LTAS Mean (dB)	11.78	17.36	15.79	14.98 (2.88)
	Alfa Ratio (dB)	−11.28	−9.32	−4.34	−8.31 (3.58)
				
Singer 3	*F*_1_ (Hz)	661.65	643.44	646.85	650.65 (9.68)
	*F*_2_ (Hz)	1365.65	1313.38	1303.22	1327.42 (33.50)
	*F*_3_ (Hz)	2782.94	2753.06	2727.45	2754.48 (27.77)
	*F*_4_ (Hz)	3639.3	3530.10	3552.19	3573.86 (57.74)
	*F*_5_ (Hz)	4520.81	4545.24	4480.63	4515.00 (32.62)
	*R*_1_:*f*_0_ Tuning	2.14	2.30	−3.97	0.16 (3.57)
	SFC (dB)	−74.26	−72.18	−74.26	−73.56 (1.20)
	Ratio H1-H2 (dB)	17.5	13.5	5.7	12.23 (5.99)
	LTAS Mean (dB)	17.84	18.78	17.84	18.15 (0.54)
	Alfa Ratio (dB)	−14.72	−12.39	−14.72	−13.94 (1.35)

Singer 1 exhibited the most concentrated resonance grouping, effectively clustering the SFC (2500–3,500 Hz). This was evidenced by a distance of only 702.7 Hz between *F*_3_–*F*_4_ (2829.24 Hz and 3531.94 Hz, respectively) and further supported by the strategic positioning of F5 at 4620.61 Hz. This resonance optimisation was facilitated by a 24.13 Hz expansion of the vowel space—increasing the – distance to 770.05 Hz during congruent induction relative to her 745.92 Hz baseline—which effectively enhanced both spectral brightness and articulatory clarity. These adjustments resulted in a robust LTAS Mean of 17.65 dB and an Alpha Ratio of −6.09 dB, providing physical evidence of enhanced spectral brilliance and ‘squillo’. Regarding the glottal source, her *H*_1_–*H*_2_ ratio of 6.5 dB indicated a balanced and healthy phonatory effort (Table 5).

In the incongruent condition, Singer 1 experienced a clear disruption in vocal control as she started from a high baseline Positive affect score of 3.00 in the pre-test condition, which decreased by 0.30 points in the post-performance measurement. Concurrently, her Tension score increased by 0.17 points, while no increases were observed for Hostility (Table 1). This emotional misalignment directly impacted her vocal source dynamics: her peak intensity (*I*__max_) decreased substantially, and the glottal source reflected instability with an *H*_1_–*H*_2_ ratio of 9.5 dB, suggesting a more lax or ‘breathy’ phonation. Furthermore, her vibrato morphology showed irregularities, with the extent falling to 106.15 cents (Table 4).

These source disruptions triggered a simultaneous breakdown in her resonance strategies, compromising the overall vocal quality. Specifically, the loss of resonant stability was evidenced by a marked increase in *R*_1_:*f*_0_ divergence, reflecting a breakdown in the harmonic–formant alignment compared to the congruent state. This was corroborated by a widespread reduction of 88.92 Hz in her vowel space (*F*_1_–*F*_2_ gap), indicating a more neutralized articulation. These adjustments led to a substantial loss of resonant efficiency: her SFC intensity dropped to −71.82 dB, her Alpha Ratio fell to −6.53 dB, and her LTAS mean decreased to 12.68 dB. Together, these metrics provide quantitative evidence of how emotional dissonance compromises the fine motor coordination and spectral brilliance observed during congruent induction (Table 5).

Singer 2 during the Joy excerpt revealed a robust technical baseline. Under congruent induction, this participant demonstrated notable emotional regulation with a mean Positive affect score of 2.70 (Table 1), calculated as the average of a pre-test score of 2.80 and a post-performance score of 2.60, reflecting a sophisticated integration of technical control rather than an involuntary psychophysiological shift. This technical robustness was manifested in a visible intensification of the upper formants and global harmonic saturation. The congruent induction directly influenced her vocal source dynamics (Table 4), enabling a peak intensity (*I_-max_*) of 75.10 dB and a *F*_0-max_ of 731.71 Hz. Her vibrato demonstrated a stable and sustained morphology, with an extent of 129.16 cents and a rate of 5.34 Hz, ensuring a vibrant vocal quality that maintained artistic consistency under congruent affect.

These source dynamics were synergistically supported by her vocal filter and resonance markers (Table 5). Singer 2 achieved high-precision alignment in her resonance strategy, evidenced by an “*R*_1_:*f*_0_ tuning” divergence of only −13.13 Hz. Her formant configuration featured an *F_1_* of 660.50 Hz and an *F_2_* of 1328.52 Hz, while her upper resonances were positioned at 2817.23 Hz (*F_3_*), 3765.73 Hz (*F_4_*), and 4625.12 Hz (*F_5_*). This configuration resulted in an optimised SFC (2500–3,500 Hz) with an intensity of −71.47 dB, manifested in a visible intensification of the upper formants and global harmonic saturation. Furthermore, her glottal source reflected a perfectly balanced phonatory state, with the *H_1_*–*H_2_* ratio shifting to 0 dB. Ultimately, these adjustments produced a robust LTAS Mean of 17.36 dB and an Alpha Ratio of −9.32 dB, providing objective evidence of the high-energy spectral distribution and professional projection required for the expression of joy.

Under the incongruent condition for the Joy excerpt, Singer 2 demonstrated notable technical resilience despite the affective dissonance. While the induction of joy remained professionally regulated, her Positive affect score fell slightly by 0.10 points, while her Hostility score rose by 0.33 (Table 1). This state of emotional misalignment suggested that while the participant experienced a minor psychological shift, her technical framework remained the primary driver of the vocal output. An analysis of her vocal source dynamics (Table 4) revealed that the congruent induction facilitated a peak intensity (*I_-max_*) reached 75.29 dB and her *F*_0-max_ was 758.61 Hz. Notably, unlike the disruptions observed in other participants under conflict, she preserved a wide and regular vibrato morphology with an extent of 133.45 cents and a rate of 5.29 Hz. This sustainment of frequency modulation provided physical evidence of her ability to maintain fine motor coordination even when artistic intent and induced affect were in conflict.

The vocal filter and resonance markers (Table 5) acted as a resilient framework, providing synergistic support that remained remarkably stable despite the dissonance. Although her resonance strategy showed an increase in “*R*_1_:*f*_0_ tuning” divergence to −18.41 Hz, her formant configuration—featuring an *F*_1_ of 671.24 Hz and upper resonances at 3640.81 Hz (*F*_4_) and 4480.07 Hz (*F*_5_)—effectively maintained the SFC (2500–3,500 Hz) at an intensity of −71.06 dB.

The glottal source reflected the underlying emotional conflict through a shift toward a more lax phonation, evidenced by her *H_1_*–*H_2_* ratio increasing to 6.4 dB. Nevertheless, her technical robustness ensured a high LTAS Mean of 15.79 dB and resulted in her most robust Alpha Ratio of −4.34 dB. Ultimately, these findings suggest that Singer 2’s superior professional regulation and optimised technical baseline enabled her to sustain resonant integrity and acoustic power throughout the state of affective dissonance.

Singer 3, under the congruent condition for the Joy excerpt, maintained a highly optimised technical foundation, which required minimal structural reconfiguration, as her non-induction baseline technique was already maximised for projection and brilliance. The emotional induction was professionally regulated but largely ineffective in terms of felt emotion, as evidenced by her Positive affect score starting at a pre-test level of 1.90 and increasing by only 0.10 points in the post-performance measurement. Concurrently, her Tension and Hostility scores began at high baseline levels of 1.83 and 1.50, decreasing by 0.16 and 0.17 points, respectively (Table 1). As evidenced by the vocal source dynamics in Table 4, the participant achieved a peak intensity (*I_-max_*) of 77.46 dB and a *F*_0-max_ of 715.78 Hz. Her vibrato remained stable and technical, displaying an extent of 146.37 cents and a rate of 5.50 Hz. These parameters underscored her reliance on a superior technical baseline to maintain artistic consistency, with affective manifestation observed only through subtle temporal modulations towards the end of the emission.

As a resonant counterpart to these source dynamics, her vocal filter and resonance markers (Table 5) exhibited a series of synergistic configurations. Singer 3’s resonance strategy was characterised by an exceptional “*R*_1_:*f*_0_ tuning,” achieving the study’s minimal divergence of only 2.30 Hz between her *F*_1_ (643.44 Hz) and the mean *f*_0_ (641.14 Hz). This precise coupling supported a robust SFC intensity of −72.18 dB and a robust LTAS Mean of 18.78 dB, providing objective evidence of the high-energy spectral distribution characteristic of her professional output. Furthermore, her glottal source reflected an energised and open phonatory state, evidenced by an *H_1_*–*H_2_* ratio of 13.5 dB. Ultimately, her Alpha Ratio of −12.39 dB further confirmed a well-balanced spectral slope. These findings highlight that for Singer 3, the expression of joy was facilitated by an intrinsically optimised vocal architecture that required no marked additional adjustments under congruent affect.

Conversely, the incongruent condition for the Joy excerpt triggered a shift in vocal production for Singer 3, as affective dissonance impacted her technical stability, with her Positive affect score decreasing by −0.30 points. While her Tension and Hostility scores increased by 0.66 and 0.34 points, respectively (Table 1). This emotional misalignment suggested that despite a robust technical baseline, the participant remained vulnerable to the tension induced by emotional dissonance. In terms of vocal source dynamics (Table 4), this physiological tension was evidenced by the vibrato pattern, which became narrower and faster, with the extent falling to 135.70 cents and the rate increasing to 5.72 Hz. During this condition, her *F*_0-max_ was 728.54 Hz, while her peak intensity reached 79.82 dB. These temporal modulations and restricted frequency oscillations provided physical evidence of the disruption to her fine motor control, undermining the expansive character of her professional emission.

These source dynamics were further impacted by changes in her vocal filter and resonance markers (Table 5). The glottal source reflected a marked increase in laryngeal constriction, with her *H_1_*–*H_2_* ratio dropping to 5.7 dB (from a congruent state of 13.5 dB), indicating a shift toward a more ‘pressed’ phonation. Quantitatively, this state was characterised by a return to baseline resonant levels, with her Alpha Ratio reaching −14.72 dB and her LTAS Mean recorded at 17.84 dB. Her resonance strategy also showed a “*R*_1_:*f*_0_ tuning” divergence of −3.97 Hz. Ultimately, these acoustic disruptions confirm that affective dissonance compromised the expansive and open character of her professional joyful emission, demonstrating that emotional conflict can destabilise even a highly optimised vocal architecture.

### Global interpretation

The efficacy of the emotional induction protocol was evidenced by distinct patterns of affective response among the participants (Table 1). While Singer 1 proved the most reactive—showing a substantial upward shift in positive affect that acted as a pronounced additive catalyst to her performance—Singers 2 and 3 exemplified superior professional emotional regulation. Their stable psychometric scores reflect a sophisticated integration of pre-established technical frameworks, allowing for intentional interpretative adaptations rather than involuntary psychophysiological shifts. This technical robustness underscores their ability to channel emotional stimuli through an expert-controlled vocal mechanism, ensuring that the ‘fertile ground’ for resonance optimisation was maintained through professional agency rather than mere emotional reactivity. Consequently, artistic consistency was preserved even under the conflicting demands of the incongruent phase.

Regarding her vocal source dynamics (Table 4), Singer 1 exhibited a distinct physiological response under the congruent condition; this reflected a robust and balanced phonatory status, where she achieved maximum projection without reaching excessive glottal tension, evidenced by a 21.82 Hz decrease in her -max. This strategy of phonatory efficiency was consistent with the results observed in Singer 2 and Singer 3, although with different degrees of intensity. While Singer 1 reached a peak intensity of 73.19 dB, Singer 2 demonstrated a similar acoustic behaviour, achieving a peak of 75.10 dB with a controlled -max (731.71 Hz). Singer 3, however, displayed the most powerful response in terms of sound pressure, reaching 77.46 dB while maintaining the lowest -max of the group (715.78 Hz). Collectively, these findings suggest that for the Joy excerpt, the singers did not rely on an upward shift of the fundamental frequency to increase their volume. Instead, the data points towards a unified technical strategy among the participants: the prioritisation of resonant reinforcement over glottal effort, allowing for higher intensity peaks with a more relaxed and efficient laryngeal configuration.

Regarding vibrato morphology (Table 4), the parameters observed provided critical qualitative insights into the singers’ technical responses. Under the congruent condition, Singer 1 exhibited a highly stable and sustained configuration with an extent of 132.87 cents, characterised by an ‘accentuated’ harmonic morphology and darker spectral peaks that reflect adaptations associated with high-arousal positive states. While this technical precision was shared by the other participants, they displayed distinct variations: Singer 2 showed a slightly narrower, more lyrical extent (129.16 cents) with a relaxed rate (5.34 Hz), whereas Singer 3 demonstrated the most expansive vibrato of the group, with a notably larger extent (146.37 cents) and a faster rate (5.50 Hz). Collectively, these findings suggest that the induction of joy facilitates a physiological state where periodic vocal fold oscillations remain uncompromised; this stands in stark contrast to the affective dissonance triggered by incongruent conditions. For instance, the incongruent state led to a general reduction in amplitude for Singer 1 (falling to 106.15 cents) and a constricted, accelerated pattern in Singer 3 (5.72 Hz rate; 135.70 cents extent), highlighting the disruptive impact of emotional conflict on fine glottal control.

The vocal filter and resonance analysis (Table 5) confirmed that Singer 1 utilised sophisticated strategies to express joy. She was the only participant to demonstrate a greater separation between *F*_1_ and *F*_2_ under the congruent condition, effectively expanding her vowel space to 770.05 Hz (increasing the *F*_2_–*F*_1_ distance by 24.13 Hz relative to baseline). This was accompanied by a consistent application of “*R*_1_:*f*_0_ tuning” with a minimal divergence of 14.96 Hz, which optimised resonance and projection. This strategy facilitated a deliberate adjustment of the vocal tract to energise the upper formant range, characterised by a precise alignment of *F*_3_ (2829.24 Hz) and *F*_5_ (4620.61 Hz) with the fourth and fifth harmonics during the central portion of the vibrato cycle. This fostered a more compact SFC, with a distance of only 702.70 Hz between *F*_3_ and *F*_4_, enhancing the “squillo” characteristic of genuine joy. While Singers 2 and 3 achieved brilliance through alternative strategies—recording SFC intensities of −71.47 dB and −72.18 dB, respectively—they exhibited levels of resonance-to-harmonic coupling comparable to the induced state of Singer 1. Finally, the incongruent condition triggered a widespread reduction in inter-formant distance (e.g., Singer 1’s gap falling to 657.00 Hz) and a decline in harmonic–formant coincidences, demonstrating that emotional conflict undermined the fine motor coordination required for stable resonance.

Regarding the incongruent condition for the Joy excerpt, the previously observed resonance-based efficiency underwent a marked decline, revealing how affective dissonance undermines fine vocal control. While the congruent state was defined by precise “*R*_1_:*f*_0_ tuning,” the incongruent induction disrupted this alignment across all participants. Singer 1 exhibited a notable contraction of her vowel space, with the *F*_1_–*F*_2_ gap narrowing substantially; this was accompanied by a reduction in spectral energy within the higher formants, suggesting that the lack of emotional ‘buoyancy’ hindered her ability to maintain a stable resonant cluster. Similarly, Singers 2 and 3 displayed a marked decrease in vibrato stability; their extent fell below baseline levels, and the morphology became irregular, reflecting a loss of the relaxed laryngeal configuration required for the ‘squillo’ effect. These findings indicate that without emotional congruence, the physiological ‘flow’ necessary for lyrical optimisation is replaced by a more effortful and less resonant phonatory state, where the singers fail to activate the acoustic markers of joy despite the technical demands of the musical score.

## Discussion

In our research, I have analysed to what extent the induced emotion modified the singing voice of professional lyrical singers relative to their neutral baselines. The results indicated that vocal features (specifically SPL, *F*_0_ and vibrato dynamics) varied consistently with the proposed emotional categories.

Analysis of the Anger/Rage musical excerpt under varying induction conditions revealed that the programme was effective for Singers 2 and 3, who manifested heightened levels of hostility and anger, whereas Singer 1 remained affectively stable. Acoustic results showed that all sopranos increased their peak sound intensity (*I*_-max_) during the congruent condition compared to the baseline. Regarding the *F*_0_, Singers 2 and 3 demonstrated a pronounced upward shift in *F*_0-max_, while Singer 1 recorded a decrease. Furthermore, in contrast to Singer 1, the participants for whom induction was effective (Singers 2 and 3) exhibited higher *F*_0_ variability and an expanded vibrato extent. These collective acoustic markers were consistently associated with the high-arousal negative activation characteristic of anger or rage within professional lyrical performance.

During the performance of the Joy musical excerpt, the emotional induction programme proved most effective for Singer 1, who exhibited markedly higher positive affect scores compared to the other participants. Acoustically, this singer was the only one to demonstrate an increase in both the maximum and minimum vibrato values during the congruent condition, even though variability remained stable. In contrast, the induction process was ineffective for Singer 3, as neither the joyful nor the negative stimuli triggered pronounced affective shifts during the performance of this fragment.

In the scientific literature, the relationships between acoustic parameters and the expression of emotions have been analyzed (Eyben et al., 2015; Hakanpää, 2022; Hakanpää, et al., 2021, 2023; Juslin and Laukka, 2003; Kamiloğlu et al., 2020; Scherer et al., 2017). Notably, these sources consistently identified SPL and *F*_0_ as the most robust indicators of high-arousal emotions. The results suggested that the expression of emotions characterised by higher levels of arousal––such as joy, fear, anger, and hostility – was associated with specific acoustic parameters, including increased singing intensity (i.e., *I*_-max_), faster tempos, and a higher rate of volume fluctuation, and, finally, an enhancement of the singer’s formant amplitude; particularly in higher frequencies. This profile contrasted sharply with emotions associated with lower arousal levels, such as sadness, tenderness, and love, which typically exhibited the opposite acoustic trends.

In our research, we analyzed to what extent the induced emotion modified the acoustic parameters of the singing voice in professional lyrical singers. Accordingly, our discussion compared the acoustic parameters of singers for whom the emotional induction process was effective versus those for whom it was not, based on the emotional musical excerpt performed.

As previously mentioned, the process of inducing Anger/Rage emotions was most effective for Singer 2 under the congruent condition. Under this condition, Singer 2 showed a notable increase in *I*_-max_ and the *F*_0-max_ variable compared to the non-induction condition. This pattern was consistent with findings by Liu et al. (2022), who pointed out that higher *F*_0_ values were typically associated with increased muscle tension and higher subglottic pressure, which were characteristic of anger. In this context, it is important to note that quantifying the in the singing voice is always an approximation, as the professional voice rarely remains stable for more than a few moments, even on the same note. This observation was further corroborated by Singer 3, who also showed an increase in *I*_-max_ and *F*_0-max_ between the non-induction to the congruent condition. Notably, Singer 3 exhibited the lowest variability across all conditions, suggesting a highly stable vocal output when expressing high-arousal emotions. These results aligned with the study by Hakanpää (2022), which indicated that high-energy emotions such as anger tended to notably elevate the base pitch.

In lyrical singing, acoustic phenomena such as “*R*_1_:*f*₀ tuning” and the “Singer’s Formant” were crucial for optimizing vocal resonance, thereby contributing to voice projection and brilliance. Several studies (Garnier et al., 2010; Joliveau et al., 2004) showed that when a singer adjusted the first vocal tract resonance (*R*1) to align closely with the F_0_ —a phenomenon known as “*R*_1_:*f*₀ tuning”— vocal efficiency was enhanced through the amplification of the first harmonic and improved acoustic projection. In the present study, we observed that in Singer 2—where the emotional induction of anger/rage appeared to be most effective—*R*_1_ did not consistently align with *f*₀, particularly during the initial phase of the vibrato cycle, where resonance–harmonic coincidences were minimal. Similarly, the degree of alignment between *R*_2_ and 2*f*₀ decreased when comparing the non-induction to the congruent condition; particularly in contrast to the other singers. This divergence in tuning may have contributed, especially in Singer 2, and to some extent in Singer 3, to a vocal output perceived as more effortful, less bright, and less acoustically defined under conditions associated with negatively valenced emotional expression. These findings were consistent with previous research indicating that high-arousal, negatively valenced emotions (such as anger or fear) were often associated with greater laryngeal tension, spectral irregularity, and reduced harmonic–formant coherence (Juslin and Laukka, 2003; Patel et al., 2011), resulting in a timbre perceived as more pressed and emotionally charged but less resonant and balanced.

Finally, in this study we observed how the induction of anger differentially modified the relationships between the SFC and the harmonics of the singers. Consequently, studying the relationship between formants and harmonics offered an important perspective for understanding how vocal performances can be influenced by emotion. In Singer 2, and to a greater extent Singer 3, the distances between the upper harmonics (i.e., *F*_3_ and *F*_4_) were broad; however, marked clarity and a high concentration of energy were observed in the higher harmonics. This suggested these singers might be achieving Singer’s Formant prominence through increased intensity or more efficient excitation of these formants, leading to very strong “projection” (Sundberg, 2001a, 2001b). Hakanpää et al. (2019, 2023) demonstrated that emotions like anger were associated with an increase in the alpha ratio (i.e., relative energy in high frequencies), which reflected a greater prominence of the *F*_3_–*F*_4_ cluster. Also, Sundberg et al. (2024) directly related the increase in spectral energy within the singer’s formant region to enhanced vocal projection and sound penetration, especially under emotional conditions such as anger. Therefore, these singers were employing an effective strategy to generate strong resonance in the singer’s formant region, contributing to their “penetrating voice,” consistent with the induction of anger/rage.

These individual variations highlight the singer-specific nature of professional vocal regulation and source-filter coordination under heightened emotional conditions. From a neuroscientific perspective, functional and structural evidence indicates that expert opera singers develop enhanced, experience-dependent neuroplasticity in singing-related auditory and somatosensory networks. Long-term vocal training leads to distinct macro-structural adaptations, including increased gray matter volume in the right somatosensory and auditory cortices, alongside specialized functional tuning in associated regions like the right anterior insula (Kleber et al., 2013, 2016, 2017; Zarate and Zatorre, 2008). Since these integrated neural networks govern fine motor coordination and modulate vocal tract posture based on sensory feedback, such deep-seated neuroplastic changes suggest that inter-individual differences in acoustic strategies are not merely superficial technical or stylistic choices. Instead, they appear to be anchored in experience-dependent brain alterations, offering a robust explanation for why professional vocalists exhibit distinct, singer-specific resilient mechanisms to maintain or adapt their resonance tuning and acoustic stability under emotional stress.

It is also highly probable that these diverse acoustic strategies are closely related to individual pitch-perception preferences and auditory-cortical asymmetries. Research indicates that vocalists differ fundamentally in their sensory processing, showing a different reliance on *F*_0_-related cues versus spectral and formant-related cues during pitch regulation (Schneider et al., 2005). Consequently, the distinct focus observed between Singer 2 and Singer 3—where one prioritises laryngeal/harmonic tension and the other manages resonance and formant clustering—could reflect neurobiological differences in individual auditory perception and sensory preferences rather than purely stylistic variations.

This sensorimotor resilience can be further contextualized within the specialized audio-vocal feedback architecture characteristic of elite vocalists. Neuroscientific investigations into pitch regulation and auditory feedback highlight a critical distinction between involuntary, emotion-driven vocal changes and the voluntary, intentional expressive control maintained by professionals (Zarate and Zatorre, 2008; Zarate et al., 2010; Zarate, 2013). While intense, felt emotions automatically modulate fundamental frequency (*F*_0_) and laryngeal stability via subcortical pathways, expert singers rely on highly trained cortical loops to monitor and correct vocal output in real time. Consequently, the acoustic shifts observed under conditions of affective congruence and incongruence in this study should not be interpreted as passive, purely acoustic consequences of emotional arousal. Instead, they reflect a dynamic interplay within a highly trained audio-vocal feedback system, where the cognitive and emotional load of professional acting and conflicting sensory conditions can either challenge or recruit voluntary sensorimotor regulation to preserve pitch accuracy and vibrato stability.

Holmes (2013) observed that intense emotions could affect vibrato stability. Expanding upon this finding, various authors (Dromey et al., 2015; Sundberg, 1994) investigated how singing with emotion influenced acoustic parameters of vibrato, such as rate, extent, and regularity. Scherer (1986) specifically noted that anger and joy were associated with high *F*_0_ variability, hypothesizing that *F*_0_ variability in speech translated into vibrato extent in singing. This suggested that emotional expression influenced both the extent and regularity of vibrato. Consistent with these findings, our results show that singers exhibited similar vibrato rates in the congruent condition. However, differences between singers and conditions were evident in the extent variable (cents), which reflected frequency oscillations. Under the congruent condition, Singer 2 primarily utilized extent amplification to intensify the expressed emotion, while Singer 3 relied on the impeccable sustain of her already optimized vibrato and harmonic density. These distinct strategies were employed to intensify the emotion of anger/rage, thereby conveying a heightened sense of energy and dramatic impact. This individual modulation and the maintaining or loss of vibrato stability and pitch accuracy under emotional load can be further explained through a neuroscientific framework of vocal control. Neuroimaging studies on trained vocalists demonstrate that elite singing relies on a highly specialized, experience-dependent integration of auditory and somatosensory feedback loops, where the right anterior insula and associated sensorimotor regions play a pivotal role (Zarate and Zatorre, 2008; Kleber et al., 2013, 2017). When intense emotional tasks or affective conflicts overload these central auditory-motor networks, the precision of fine motor coordination can be disrupted, explaining the distinct singer-specific fluctuations observed in variables like vibrato extent. This finding further reinforced the notion that emotional engagement directly impacted vibrato characteristics. Supporting this perspective, Dromey et al. (2015) found that vibrato rate was specifically associated with higher emotional load, and vibrato extent was greater in more expressive passages. Both vibrato parameters appeared to be involved when singers expressed emotions with high activation (such as joy or anger/rage) (Juslin and Laukka, 2003; Ternström, 2003). In fact, Singer 1, where the emotional induction process was not effective, she exhibited minimal changes in her vibrato, which underscored the importance of emotional activation in the acoustic manifestation of expressive intention in lyrical singing.

As previously discussed, the acoustic analyses in this study revealed distinct individual patterns in the vocal expression of anger among the three operatic singers, even while sharing some general characteristics associated with emotional arousal. Consistent with prior research, all participants exhibited an increase in high-frequency energy and spectral noise under congruent induction (Bugdol et al., 2024; Juslin and Laukka, 2003; Ponsot et al., 2017; Scherer, 2003). Fundamentally, these findings demonstrate that interpretive mastery is an enactive process, wherein the performer’s ‘emotional mind’ does not merely represent an affect externally, but rather embodies it through a real and profound biomechanical reconfiguration (Leman, 2016; Schiavio and van der Schyff, 2018). This phenomenon is grounded within the paradigm of 4E/5E Cognitive Science, suggesting that the induced affective state acts as a catalyst that triggers involuntary, neurovegetative, and somatic responses (Johnstone and Scherer, 2000; Scherer, 2009). These responses physically alter subglottic pressure, laryngeal muscular tension, and the geometric configuration of the vocal tract (Klasen et al., 2013; Sundberg, 1974), ensuring that the analysed acoustic markers reflect authentic physiological processing rather than a mere conscious technical simulation (Juslin and Laukka, 2003).

Specifically, Singer 1 exhibited the lowest level of subjective anger during the induction process, confirming that the protocol was ineffective for this particular emotion in her case. Consistent with this lack of affective activation, acoustic analyses demonstrated lower acoustic tension, characterised by the maintenance of a highly regular vibrato and a relatively low dispersion of high-frequency energy. This evidences a remarkable capacity for vocal control, wherein the absence of a genuine emotional substrate forced the performer to rely exclusively on trained, automated vocal mechanisms to simulate the demanded affect (Scherer and Sundberg, 2017). Rather than displaying a spontaneous biological response, this singer employed a strategy of aesthetic representation or conscious technical simulation of anger, attempting to compensate for the lack of induction through a voluntary increase in glottal tension to provide projection and brilliance, whilst preserving a strictly regular and controlled vocal resonance. From a post-cognitive perspective, the psychophysiological stability of Singer 1 demonstrates that, in the absence of genuine affective induction, the performer is deprived of the necessary somatic pre-activation, operating instead from an expert yet cognitively distanced control mechanism. In this scenario, technical execution substitutes for the authentic embodiment of emotion, resulting in a formally correct performance that is nonetheless devoid of the involuntary and enactive biomechanical reconfiguration that defines organic expressive excellence under conditions of high emotional intensity (Colombetti, 2014; Leman, 2016; Schiavio and van der Schyff, 2018).

Conversely, Singer 2, who showed greater variability in her mood scores and reported the highest levels of subjective anger, exhibited the most acoustically perturbed vocal output. This was evidenced by a reduction in vibrato regularity, unstable formant trajectories, and a diffuse distribution of upper harmonic energy—factors linked to a more abrupt and angular glottal source waveform—resulting in a perceptually ‘harsh’ or ‘strained’ vocal quality. This outcome indicates that heightened subjective emotional intensity can lead to increased physiological tension that compromises fine motor control and vocal efficiency, manifesting as greater perceived vocal effort (Bugdol et al., 2024; Ponsot et al., 2017). Under the 4E/5E Cognitive Science framework, this profile suggests that affective induction triggered somatic and involuntary responses so potent that they challenged the singer’s sensorimotor coupling. The intensity of the ‘felt’ affect caused an overload in the auditory-motor and somatosensory control networks (Zarate and Zatorre, 2008), demonstrating that the process of emotional enaction can destabilise technical consistency if the psychophysiological load temporarily overwhelms the performer’s capacity for self-regulation (Colombetti, 2014; van der Schyff et al., 2018).

Finally, Singer 3 initially presented with elevated negative affect scores, subsequently reporting high levels of subjective anger and psychological tension during the emotional induction. Her vocal manifestation was distinct, characterised by a pronounced increase in high-frequency energy and spectral noise. Notably, while the persistent absence of ‘R1:f0 tuning’ suggested a loss of primary resonance efficiency, this was strategically compensated by a marked elevation in raw Sound Pressure Level (SPL) and the robust reinforcement of the upper formants. This concentration of energy within the SFC, combined with a wide vibrato extent that remained rhythmically stable, facilitated a highly efficient, albeit intensive, vocal tract modulation. This mechanism allowed her to channel anger into a powerful, penetrating vocal quality without a substantial loss of harmonic integrity (Ponsot et al., 2017). Such a configuration represented a controlled and directed expression of rage, leveraging strong glottal compression to achieve dramatic acoustic impact while preserving the essential qualities of the lyrical voice (Scherer and Sundberg, 2017). Singer 3 epitomises the ultimate expression of interpretive mastery as an enactive process: the capacity to coexist with a genuine ‘physiological tempest’ and subjugate it to optimise acoustic output. By mastering an involuntary biological response of anger and transforming it into a potent, stable acoustic signal, the singer demonstrates that her emotional mind drives an authentic biomechanical reconfiguration, ensuring that her acoustic markers reflect real, embodied physiological processing rather than a mere conscious technical simulation (Scherer and Sundberg, 2017).

The discussion of the Joy musical excerpt begins with the observation that emotional induction was most effective for Singer 1 in the congruent condition. Interestingly, despite this effective affective activation, Singer 1’s vocal intensity (*I*_-max_) did not show a notable increase compared to the non-induction condition. However, a critical finding was the pronounced reduction in her *F*_0-max_ compared to her baseline condition, which converged closely with the nominal pitch of the high-pitched target note. Whilst previous literature (Juslin et al., 2014; Juslin and Laukka, 2003; Scherer, 2003) suggests that high-arousal emotions like joy typically trigger an upward shift in fundamental frequency, Singer 1’s data reveals a more sophisticated physiological adaptation. By lowering the *F*_0_ peak towards the nominal frequency of the note whilst maintaining a robust sound pressure level, she demonstrated a transition towards ‘flow phonation’. This suggests that the induction of joy facilitated a more efficient coupling between the glottal source and the vocal tract, where resonant reinforcement—evidenced by her precise ‘*R*_1_:*f*_0_ tuning”—obviated the need for compensatory glottal tension. This technical robustness underscores how emotional congruence facilitates laryngeal economy in expert performers, allowing them to achieve high-arousal positive expressions through resonance optimisation rather than increased physiological effort (Scherer and Johnstone, 2003).

This remarkable laryngeal economy correlates directly with the active modulation of formant variables, confirming that the articulatory and resonance configurations of the vocal tract are dynamically altered by the expressed emotional state (Goudbeek et al., 2009; Mendes A. P. et al., 2003; Mendes F. et al., 2003; Waaramaa-Mäki-Kulmala, 2009). Specifically, the filter reconfiguration observed in Singer 1 supports the findings of Sundberg (2021), who demonstrated that professional opera singers systematically modulate acoustic parameters to convey distinct emotional affects. In this case, the efficient convergence between harmonic partials and upper formant frequencies—evidenced by her precise resonance tuning and a marked expansion of the vowel space—provided the necessary acoustic scaffolding. This resonance strategy effectively clustered the SFC by aligning the third (*F*_3_) and fifth (*F*_5_) formants, which dramatically enhanced the spectral brightness, clarity, and *squillo* of the voice. Furthermore, this condition displayed a highly accentuated harmonic morphology from the very onset of the emission, characterised by greater stability, darkness, and prominence in the harmonic peaks. In line with the observations of Morozov (1996) and Scherer and Sundberg (2017), this configuration highlights how phonatory adaptations associated with genuine positive affect contribute to overall spectral richness, actively facilitating the listener’s neural processing of emotional musical communication.

Singer 1 epitomises the ultimate expression of interpretive mastery as an enactive process: the capacity to translate an authentic internal experience of joy into an optimal acoustic architecture. By mastering the physiological activation of positive affect and transforming it into a highly efficient acoustic signal, the singer demonstrates that her emotional mind drives an authentic biomechanical restructuring of the vocal apparatus. From a post-cognitive 4E/5E perspective, this geometric optimization of the vocal tract did not function as an isolated mechanical device. Instead, it represents the embodied and enactive realization of a positive affective state, demonstrating how a felt emotion can construct a temporary ‘affective niche’ that actively liberates the laryngeal mechanism from redundant mechanical strain (Colombetti, 2014; Schiavio and van der Schyff, 2018; Scherer and Sundberg, 2017).

In operational terms, these acoustic outcomes confirm that interpretive mastery within positive-valence conditions manifests precisely through this enactive and embodied process, wherein the performer’s ‘emotional mind’ does not merely simulate an affect through top-down cognitive mechanisms, but rather instantiates it via a global biomechanical reconfiguration. Grounded within the paradigm of 4E/5E Cognitive Science, the successfully induced state of joy operates as a somatic catalyst that modulates the neurovegetative baseline of the vocal apparatus (Scherer, 2009). This structural coupling allows Singer 1 to dynamically reconfigure vocal tract geometry and subglottic pressure reflexively, substantiating the formation of the aforementioned ‘affective niche’ (Colombetti, 2014) through the facilitation of an optimal resonance balance (*R*_1_:*f*_0_). Consequently, authentic emotional activation liberates the peripheral motor system from compensatory or redundant mechanical strain, achieving a state of physiological synergy that serves as a benchmark of elite aesthetic virtuosity.

In contrast to the organic, embodied manifestation observed in Singer 1, the acoustic behaviours of Singer 2 and Singer 3 under the same joy induction protocol reveal a completely different interpretive framework, primarily due to the subjective inefficacy of the emotional induction in their cases. For Singer 2, the transition to the congruent condition did not stem from an internal affective state, yet her data revealed an increase in harmonic density and an intensification of the upper resonances within the *F*_3_ and *F*_4_ regions. These deliberate adjustments allowed her to enhance projection and timbral brilliance, effectively portraying joy through a volitional maximisation of the Singer’s Formant Cluster. From a post-cognitive 4E/5E perspective, these modifications must be interpreted as a purely conscious technical strategy or a pre-established interpretive device rather than an enactive phenomenon. The vocalist deployed her highly trained motor resources to cognitively simulate the requested affect, operating through a top-down, disembodied mechanism. This contrasts sharply with Singer 1, whose spectral adjustments emerged as an involuntary, integrated consequence of genuine emotional arousal. In this scenario, the absence of an authentic affective niche (Colombetti, 2014) forced Singer 2 to rely on an intellectualised representation of joy. This confirms that when the performer’s emotional mind is not structurally coupled with a real physiological catalyst, technical execution functions as an artificial substitute for authentic embodiment (Schiavio and van der Schyff, 2018; Scherer and Sundberg, 2017).

Meanwhile, for Singer 3, the acoustic variations between the non-induction baseline and the congruent conditions were minimal across all evaluated spectral and temporal parameters. Her global harmonic density, upper formant prominence, and vibrato characteristics remained virtually unchanged. Given that the induction protocol failed to generate subjective joy in this participant, this total absence of acoustic shift is highly coherent with 4E/5E tenets. Unlike Singer 2, who chose to technically simulate the emotion through volitional motor control, Singer 3 maintained her baseline execution intact, reflecting a total absence of somatic pre-activation. This finding underscores that Singer 3’s voice intrinsically possessed an exceptionally optimized level of baseline projection, leaving virtually no margin or necessity for further technical modifications in the absence of an internal emotional catalyst. Ultimately, this collective divergence highlights how the psychological efficacy of emotional induction modulates acoustic execution, clearly delineating the boundary between an enactive emotional manifestation driven by an embodied affective state (Singer 1) and a conscious technical simulation or baseline maintenance resulting from a cognitive detachment from the target affect (Singers 2 and 3) (Colombetti, 2014; Schiavio and van der Schyff, 2018).

## Conclusion

The present study analysed the extent to which induced emotions modify the acoustic parameters of lyrical singers’ singing voice. The effective emotional induction of high-arousal states, such as Anger/Rage, generated distinctive acoustic modifications, notably an increase in Sound Pressure Level (i.e., SPL) and *F*_0-max_. These changes could be associated with heightened muscle tension and increased subglottal pressure. Vibrato dynamics were also markedly altered, manifesting through either an amplified extent or the optimised maintenance of regularity and harmonic density. Regarding resonance, the absence of “*R*_1_:*f*₀ tuning” likely contributed to a perceived vocal quality that was more strained and less brilliant. However, singers employed effective strategies to maintain strong resonance within the SFC region, resulting in a “penetrating” quality consistent with the intended emotion. Collectively, these acoustic modulations serve to intensify the emotional delivery, conveying greater energy and dramatic impact during performance.

The analysis of the musical fragment “Joy” under effective emotional induction revealed key acoustic modifications in the lyrical voice. Although vocal intensity (SPL/*I*_-max_) was not the primary driver of change, the expression of high-arousal states, such as Joy, is consistently associated with elevated *F*_0_ values. Vibrato modulation emerged as a defining feature, characterised by a marked amplification in both its extent and harmonic density from the onset of phonation. This suggests a dynamic vocal adaptation where the voice acquires “vibrant,” “direct,” and “expansive” qualities, aligning with the genuine expression of Joy. Furthermore, the study confirms the importance of resonance phenomena such as “*R*_1_:*f*₀ tuning,” which efficiently optimises vocal projection, and the SFC, whose prominence enhances clarity and brilliance. These acoustic adjustments are essential for conveying Joy through a direct attack and an expansive, vibrant, and resonant character.

### Limitations

Several limitations are recognize. The most evident is the small sample size (N = 3), which means that the results are exploratory in nature and cannot be generalized beyond these three singers. Furthermore, “emotional expression” in singing cannot be interpreted as a purely spontaneous outpouring, as performers must balance felt emotion against the requirements of accurate technical precision (Connolly and Williamon, 2004). Although the term traditionally encompasses both spontaneous and acted expressions (Juslin and Sloboda, 2010), data derived from simulated performances might not be identical to spontaneous emotional events (Diego et al., 2006).

Secondly, interpreting melodies with potentially contrasting emotions might have appeared musically “unnatural”; nevertheless, this design was vital for ensuring internal validity. By keeping the melody consistent, any observed acoustic modulations or judgements could be specifically attributed to the singer’s expressive intention (Juslin and Sloboda, 2010). Finally, uncontrolled individual differences, such as specific voice timbre (Siegwart and Scherer, 1995) or a singer’s inherent ability to convey affect (Coutinho et al., 2014), might have influenced results. Due to funding constraints, the current study was unable to control for these variables by increasing the participant cohort.

### Applicability and future directions

These findings collectively underscored that the relationship between subjective emotional experience and its objective acoustic correlates is non-linear and highly individualised in trained vocalists (Thompson and Russo, 2007). The diverse acoustic strategies employed to manage emotional load—ranging from the mitigation of low-intensity anger to disruptive tension or potent constriction—highlighted the complex interplay between affective states, physiological responses, and learned vocal control mechanisms. Therefore, a comprehensive understanding of emotional vocal expression necessitates the simultaneous consideration of subjective measures and detailed acoustic analyses in conjunction with individual performer characteristics (Juslin and Laukka, 2003; Scherer, 2003).

The results suggest substantial avenues for practical application, particularly within singing-voice pedagogy. The development of systems capable of acoustically measuring a singer’s voice in real-time holds promise for providing immediate feedback, enabling performers to refine their technique and enhance their expressive skills (Coutinho et al., 2014). This is especially relevant in styles such as opera, where conveying specific affective connotations is a fundamental prerequisite (Siegwart and Scherer, 1995). Furthermore, these insights support the notion that singing-voice training specifically focused on acoustic parameters could markedly improve tonal quality and resonance, directly influencing the emotional perception of singing. This approach aligns with recent pedagogical research advocating for explicit instruction in modulating acoustic features to enhance emotional expression (Hakanpää et al., 2021; Trepo, 2012), as well as neuroscientific evidence demonstrating that targeted auditory and vocal training can induce significant short-term plasticity in neuro-auditory processing (Schneider et al., 2022). Utilizing explicit parameter-modulation training and real-time acoustic feedback systems in the studio provides a solid neuroscientific basis for advanced vocal pedagogy. By consciously training singers to stabilize and modulate specific acoustic variables under stressful or emotionally incongruent conditions, educators can directly exploit these neuroplastic mechanisms to reinforce professional vocal resilience and accelerate specialized sensorimotor learning.

## Data Availability

The original contributions presented in the study are included in the article/supplementary material, further inquiries can be directed to the corresponding author.
